# Unraveling the Genetic Diversity and Phylogeography of the “King of Vitamin C” Fruit (*Rosa roxburghii* Trattinnick) in Chinese Southwest

**DOI:** 10.1002/ece3.71369

**Published:** 2025-05-07

**Authors:** Shanshan He, Feng Pan, Chunxue Jiang, Jian Feng, Cai Zhao, Jian Jian Wang

**Affiliations:** ^1^ College of Life Sciences Guizhou University Guiyang China; ^2^ Key Laboratory of Plant Resource Conservation and Germplasm Innovation in Mountainous Region (Ministry of Education) Guizhou University Guiyang China

**Keywords:** climate change, conservation, genetic diversity, phylogeography, *Rosa roxburghii* Trattinnick

## Abstract

The southwestern region of China is one of the last repositories of biodiversity. Exploring the genetic diversity and phylogeographical structure of representative species in this area can not only provide insights into their evolutionary history but also help us specify protection plans. We conducted extensive sampling and combined ISSR, cpDNA (*psb*A‐*trn*H, *atp*F‐*trn*H, *trn*L‐*trn*F, *acc*D‐*psa*I, *trn*G‐*trn*S), and single‐copy nuclear gene (*ncpGS* and *GAPDH*) data with the MaxEnt model to investigate the genetic diversity, phylogeographic pattern and potential distribution of *Rosa roxburghii* Trattinnick, the “King of Vitamin C” fruit, in this region. The study revealed high genetic diversity within *R. roxburghii* populations, with Guizhou Province identified as the center of origin. At least two glacial refugia were presented during the Quaternary ice age. The research emphasizes the impact of climate change and geographic isolation on genetic differentiation. This study contributes to the understanding of biodiversity in the southwestern region of China and offers a new perspective on the conservation of wild *Rosa roxburghii* and other plant resources.

## Graphical abstract

The study revealed high genetic diversity within *R. roxburghii* populations, with Guizhou Province identified as the center of origin and distribution. At least two glacial refugia were present during the Quaternary ice age. The potential distribution model suggests that the suitable habitat for *R. roxburghii* may expand with future climate warming, which is significant for conservation planning. This study contributes to the understanding of biodiversity in the southwestern region of China and offers a new perspective on the conservation of wild plant resources.

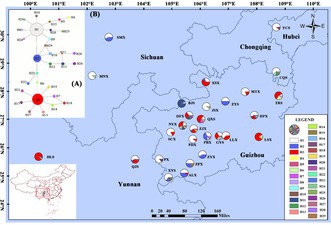

## 1 Introduction

The genetic diversity and structural patterns of plant populations are determined by processes operating at various spatial and temporal scales, which are driven not only by their life history and ecological characteristics but also by historical events such as mountain uplift and climate fluctuations associated with glacial oscillations (Comes and Kadereit 1998; Zhou et al. 2014). Complex topography provides habitats for ecological diversity, allowing organisms to find suitable niches through vertical migration during periods of adverse climatic conditions, thereby reducing the risk of extinction (He and Jiang 2014). Pleistocene climate fluctuations have had a profound impact on the genetic diversity and geographical structure of many species, causing repeated changes in their distributions (Hewitt 1996). In regions with complex climates and topography, such as southwestern China, these impacts may be more intricate. Southwestern China is one of the most important biodiversity hotspots (Wang et al. 2024), where the complex interplay between topography, climate change, and ecological factors in the dry and hot valley regions may provide a relatively stable model for ecological diversity habitats and glacial refuges. Recently, humans have further regulated and profoundly altered the climate and habitats of southwestern China through long‐term, dynamic activities, already causing and continuing to cause inestimable losses to biodiversity (Dai et al. 2008). To better understand the patterns of genetic diversity and the underlying mechanisms in southwestern China, more systematic biogeographical research is needed, which will aid in species conservation.

Neutral processes such as genetic drift, population fluctuations, inbreeding, and reduced gene flow, as well as fragmentation due to natural and anthropogenic factors, can influence the genetic diversity and structure of populations (Qiu et al. 2011; Liu et al. 2012), and systematic biogeography has recently emerged as one of the primary research methodologies. By integrating markers with different mutation rates, systematic biogeographical studies can reveal how past and present processes regulate the genetic patterns of populations (Wang 2011). Increasing evidence suggests that inconsistencies between different genetic loci are common, thus necessitating the use of multilocus genetic data to address species' formation histories (Toews and Brelsford 2012). Analyzing multilocus datasets aids in comprehending the stochastic inheritance and mutation of genes within populations, as well as comparing the genetic diversity and distribution patterns of species across various regions. This approach enables a thorough evaluation of the long‐term effects of stochastic factors on genetic diversity and structure (Brito and Edwards 2009). In recent years, the development of diverse molecular marker technologies has led to the emergence of ISSR markers, which have evolved from SSR techniques and integrated the strengths of RAPD, becoming a focal point in research. Despite limitations in reproducibility and automated analysis, the ISSR technique retains unique value in specific research domains due to its high efficiency, rapidity, stability, ease of manipulation, and cost‐effectiveness (Esselman et al. 1999; Qian et al. 2001). It has been extensively applied in the genetic diversity studies of wild species and woody plants (Zhang et al. 2021). Utilizing markers with different dispersal modes, such as chloroplast DNA (for seed dispersal) and nuclear DNA (for both seed and pollen dispersal), can quantify the relative contributions of seed and pollen flow to the genetic structure of populations (Petit et al. 2005). After considering the impact of systematic biogeographical methods on population genetic diversity and structure, we further utilized species distribution models (SDMs) to predict the distribution of species during different geological periods and in the future, aiding in the understanding of species' responses to climate change and historical events, even in cases where distribution data is incomplete (Svenning et al. 2008; Elith et al. 2010), among which the maximum entropy (MaxEnt) model is currently the most widely used niche model (Ahmed et al. 2015). By combining the MaxEnt algorithm in SDMs with molecular marker techniques, a wealth of genetic information can be provided. This approach not only facilitates the reconstruction of diversification patterns but also allows for an exploration of the responses of various species to climate change. Additionally, it enables the comparison of different marker methods to gain a deeper understanding of the dynamics of historical events and population genetic structures (Emerson and Hess 2014; Wang 2023).

Previous research has enhanced our understanding of the systematic biogeographic patterns in the southwestern region of China, largely attributed to the complex topography and climatic history (He et al. 2016; He and Jiang 2014). However, due to differences in species' dispersal abilities and habitat affinities, each species exhibits unique responses (He and Jiang 2014). Therefore, studying the genetic patterns of other endemic species remains necessary. This study selected a plant belonging to the family Rosaceae, *Rosa roxburghii* Trattinnick, as the subject of research. This plant, native and extensively distributed in the southwestern region of China, is renowned for its fruit with high content of vitamin C, superoxide dismutase, and flavonoids, and acclaimed as the “King of Vitamin C” (Fan et al. 2021a, 2021b). It is rich in nutrients, safe for consumption, and traditionally used in Chinese food and medicine. *R. roxburghii* products have been extensively applied in the food, pharmaceutical, health product, and daily chemical industries, with broad market prospects (Xu et al. 2019; Jain et al. 2024). Current research primarily concentrates on the medicinal and food product value of *R. roxburghii* germplasm resources (Zhai et al. 2023; Shen et al. 2023; Chen et al. 2023). In terms of genetic diversity, previous studies have utilized RAPD molecular markers (Wen et al. 2003), chloroplast genes combined with nuclear gene fragments (ITS and GAPDH) (Deng et al. 2015), ISSR markers (Chen et al. 2017), and EST‐SSR markers (Yan et al. 2015; Lu et al. 2020) to analyze *R. roxburghii* , laying the foundation for a deeper understanding of its genetic diversity and genetic structure. Despite the progress made by previous studies, there are still some issues in the research on the genetic diversity and systematic biogeography of *R. roxburghii* . Firstly, there are deficiencies and a relatively narrow regional scope in sample collection. Secondly, the study of the genetic background is limited by the limited genetic information provided by the markers used, failing to elaborate in detail on the genetic diversity, geographic distribution patterns, and historical dynamics of populations of *R. roxburghii* . Moreover, as socioeconomic development progresses, human activities increasingly disrupt and damage the ecological environment, leading to fragmentation in the growth of wild *R. roxburghii* , destruction of high‐quality germplasm resources, and a significant loss of genetic diversity in *R. roxburghii* resources. This underscores the imperative need for the collection, assessment, and conservation of *R. roxburghii* resources.

Our team's previous research based on two single‐copy nuclear genes and three chloroplast genes indicated that the genetic diversity of *R. roxburghii* is relatively low, with genetic variation mainly originating from within populations (Wu et al. 2023). Additionally, cpDNA is maternally inherited, has a slow mutation rate, low gene flow, and does not undergo recombination during genetic processes, making it suitable for phylogenetic and evolutionary studies (Comes and Kadereit 1998; Li et al. 2021). Adding cpDNA fragments can construct a more detailed haplotype network map, revealing a more complex population structure; it can also optimize population genetics and ensure more accurate historical inferences of the population. ISSR, due to its simple operation, good stability and repeatability, and higher fragment polymorphism, plays an important role in the research of genetic diversity and phylogenetics (Qian et al. 2006). In this study, we collected 364 specimens of *R. roxburghii* across its distribution range in southwestern China and the adjacent areas. Based on previous studies, we added a population (ZYS), two cpDNA fragments, and seven ISSR primers. We relied on the newly obtained ISSR and two cpDNA fragment datasets and combined them with the previously published data (two single‐copy nuclear genes and three cpDNA fragments, Wu et al. 2023) to compare different molecular methods and comprehensively analyze the genetic diversity and systematic biogeography of wild *R. roxburghii* . Additionally, we employed the maximum entropy model (MaxEnt) from species distribution models to predict the potential distribution of *R. roxburghii* in the southwestern region of China. Our objectives are as follows: (1) to assess the genetic diversity and population structure of *R. roxburghii* populations and to explore the potential factors influencing the genetic patterns of the species; (2) to compare different molecular marker methods to understand historical events; (3) to discuss the distribution centers of *R. roxburghii* and to grasp the overall geographical distribution pattern; (4) to predict the potential distribution of *R. roxburghii* in the southwestern region of China, providing a scientific basis for its conservation and sustainable utilization.

## 2 Materials and Methods

### 2.1 Sampling and DNA Extraction

In the southwestern region of China and its adjacent areas, a total of 364 wild *R. roxburghii* specimens were collected from 28 sites. Compared with previous studies, we have added a population of ZYS (Wu et al. 2023; Table 1), with the study sites selected to represent the various distribution areas of *R. roxburghii* and their ecological characteristics as comprehensively as possible. The collection sites were georeferenced using GPS to record their latitude, longitude, and elevation data. *Rosa cymosa* Tratt, a closely related species, was designated as the outgroup. The specimens are preserved in the herbarium of the botanical group at Guizhou University, and the corresponding voucher specimen codes are listed in Table 1. Total genomic DNA was extracted from approximately 30 mg of dry leaf tissue using a plant genomic DNA kit (Tiangen Biotech Co. Ltd., Beijing, China), as per the manufacturer's instructions, and the quality of the total DNA was evaluated using a 1.5% agarose gel electrophoresis method.

**TABLE 1:** Details of sampled populations of *Rosa roxburghii* Tratt. in southwestern China and the number of chloroplast genes (cpDNA) and single‐copy nuclear genes (SCNG) haplotypes in each population.

Pop. ID	Locality	*N*	Voucher specimen	Altitude (m)	Longitude	Latitude	cpDNA haplotypes (individual number)	SCNG haplotypes (individual number)
QXS	QianXiShi, Guizhou	14	PanCL1	1200	106.12	27.05	H1(2), H3(8), H7(2), H13(1), H14(1)	C1(7), C2(4), C3(3)
JSX	JinShaXian, Guizhou	13	PanCL2	1100	106.44	27.46	H1(12), H25(1)	C1(10), C5(2), C10(1)
DFX	DaFangXian, Guizhou	14	PanCL4	1500	105.36	27.09	H1(5), H3(4), H11(2), H12(1), H26(1), H27(1)	C1(12), C10(2)
NYX	NaYongXian, Guizhou	15	PanCL 6	1420	105.38	26.78	H1(3), H3(5), H6(1), H8(1), H11(2), H14(1), H21(1), H28(1)	C1(12), C10(3)
SCX	ShuiChengXian, Guizhou	12	PanCL7	1700	104.96	26.55	H1(10), H3(2)	C1(9), C4(2)
PX	PanXian, Guizhou	15	PanCL8	1750	104.47	25.71	H1(13), H20(2)	C1(14), C10(1)
XYS	XingYiShi, Guizhou	15	PanCL9	1250	104.90	25.08	H1(9), H2(2), H10(2), H12(1)	C1(12), C4(1), C10(2)
ALX	AnLongXian, Guizhou	14	PanCL10	1350	105.44	25.09	H1(7), H2(6), H3(1)	C1(11), C4(3)
ZFX	ZenFengXian, Guizhou	6	PanCL11	1050	105.65	25.38	H1(3), H2(2), H29(1)	C1(4), C4(1), C10(1)
ZYX	ZiYunXian, Guizhou	12	PanCL12	1165	106.08	25.75	H1(7), H2(5)	C1(10), C4(2)
PDX	PuDingXian, Guizhou	12	PanCL13	1250	105.72	26.29	H1(10), H3(1)	C1(8), C4(2), C5(2)
ZJX	ZhiJinXian, Guizhou	14	PanCL3	2200	105.77	26.66	H1(8), H2(1), H3(3), H4(1), H5(1)	C1(9), C4(3)
PBX	PingBaXian, Guizhou	12	PanCL14	1300	106.26	26.41	H1(4), H3(5), H6(1), H7(1), H8(1)	C1(7), C5(2), C7(2)
GYS	GuiYangShi, Guizhou	12	PanCL15	1050	106.69	26.33	H1(4), H3(4), H6(1), H8(1), H9(1), H10(1)	C1(10), C5(2)
LLX	LongLiXian, Guizhou	11	PanCL16	1600	106.88	26.31	H1(7), H3(4)	C1(9), C5(2)
TRS	TongRenShi, Guizhou	12	PanCL17	1700	108.45	27.49	H1(1), H3(7), H6(3), H8(1)	C1(6), C8(3), C9(3)
XSX	XiShuiXian, Guizhou	10	PanCL18	1100	106.20	28.33	H1(3), H2(1), H3(6)	C1(7), C6(3)
BJS	BiJieShi, Guizhou	16	PanCL4	1510	104.94	27.15	H1(1), H2(1), H11(13)	C1(10), C4(3), C6(2)
ZYS	ZunYiShi, Guizhou	10	GZC19	1028	106.89	27.64	H1(5), H2(5)	C1(7), C5(1), C6(1), C7(1)
MTX	MeiTanXian, Guizhou	5	PanCL19	950	107.48	27.77	H1(4), H3(1)	C1(5)
SMX	ShiMianXian, Sichuan	18	C22	20,000	101.55	29.91	H1(10), H2(8)	C1(16), C6(2)
CQS	ChongQing	18	C24	1600	108.76	28.85	H1(11), H9(2), H12(2), H15(2), H16(1)	C1(14), C5(3), C11(1)
LSX	LeiShanXian, Guizhou	10	PanCL21	1800	108.07	26.38	H1(1), H3(8), H14(1)	C1(9), C9(1)
YCS	YiChangShi, Hubei	17	C27	1600	110.93	31.08	H1(15), H3(1), H20(1)	C1(16), C9(1)
HPX	HuangPingXian, Guizhou	16	PanCL20	800	107.90	26.90	H1(9), H2(1), H3(4), H22(1), H23(1)	C1(11), C8(2), C9(2), C12(1)
DLS	DaLiShi, Yunnan	18	C26	1900	100.22	25.59	H3(16), H19(1), H20(1)	C1(9), C4(9)
MNX	MianNingXian, sichuan	10	C23	1800	102.25	27.88	H1(9), H24(1)	C1(10)
QJS	QuJingShi, Yunnan	13	C25	20,000	103.79	25.48	H1(7), H2(1), H3(4), H17(1)	C1(13)

### 2.2 ISSR Molecular Marker Analysis

Referring to the design of 100 ISSR primer sequences developed and published by the University of British Columbia (UBC), we had Beijing QingKe Biotechnology Co. Ltd. synthesize all 100 of these primers. Following preliminary trials for the ISSR‐PCR reactions of *R. roxburghii* , we selected seven primers from this collection for further experimentation (Table S1). The ISSR‐PCR reaction system and amplification protocol were modified based on the methods of Chen et al. (2017) and Wang et al. (2009). The reaction mixture, 20 μL in volume, included: 2 × Taq PCR Master Mix 9.5 μL; 10 μmol/L each of forward and reverse primers 1 μL; 40 ng/μL DNA 1 μL; ddH2O 7.5 μL. The PCR amplification procedure is as follows: initial denaturation at 94°C for 4 min; denaturation at 94°C for 1 min, primer annealing for 45 s, extension at 72°C for 1 min, for 36 cycles; final extension at 72°C for 1 min, and storage at 12°C. The amplified products were analyzed by 2% agarose gel electrophoresis, with the seven ISSR primers exhibiting high polymorphism, good stability, and bright bands. The gel electrophoresis images for each primer are presented in Figure S5.

Based on the PCR amplification results of ISSR molecular markers, the 2000 bp DNA ladder marker in the electrophoresis profile was used as a reference to manually tally the number of electrophoresis bands. Amplified bands with identical relative molecular masses and positions were considered the same locus. The presence or absence of amplification bands for each sample was recorded as “1” or “0”, respectively, to construct a data matrix. The POPGENE (Yeh et al. 1997) was utilized to calculate the total genetic variation (*Ht*), within‐population genetic variation (*Hs*), genetic differentiation coefficient (*Gst*), Nei's genetic diversity (*H*), effective number of alleles (*Na*), observed number of alleles (*Ne*), and Shannon's information index (*I*). The NTSYS (Rohlf et al. 1992) was utilized for conducting UPGMA cluster analysis to assess genetic consistency. The GenAlex (Peakall and Smouse 2012) was employed for molecular variance analysis (AMOVA), principal coordinate analysis (PCoA), and to perform Mantel tests to examine the correlation between genetic and geographical distances among populations. The MEGA (Tamura et al. 2013) was used to construct a phylogenetic tree based on the genetic distance matrix.

### 2.3 Analysis of cpDNA and scnDNA Molecular Markers

Following preliminary experiments, five published chloroplast gene intergenic spacer fragments, namely *psb*A‐*trn*H (Kress et al. 2005), *atp*F‐*trn*H (Lahaye et al. 2008), *trn*L‐*trn*F (Taberlet et al. 1991), *acc*D‐*psa*I (Small et al. 1998), and *trn*G‐*trn*S (Shaw et al. 2007), and two single‐copy nuclear gene fragments, *ncpGS* (Emshwiller and Doyle 1999) and *GAPDH* (Joly et al. 2006) (Table S1), were selected for amplification and sequencing of a total of 364 individuals from 28 *R. roxburghii* populations. The PCR amplification reaction system consisted of 25 μL: 2 × Taq PCR Master Mix 12.5 μL; 10 μmol/L each of forward and reverse primers 1 μL; 40 ng/μL DNA 1 μL; ddH2O 9.5 μL. The PCR amplification conditions were as follows: initial denaturation at 94°C for 5 min; denaturation at 94°C for 45 s, primer annealing for 40 s, extension at 72°C for 2 min, for 34 cycles; final extension at 72°C for 10 min, and storage at 12°C. The PCR products were detected by 2% agarose gel electrophoresis, and samples with clear, single, and bright bands on the gel imaging system were sent to Beijing QingKe Biotechnology Co. Ltd. for purification and bidirectional sequencing.

After obtaining the sequences and examining the chromatograms, we aligned the sequences using the ClustalW algorithm in MEGA (Tamura et al. 2013) and BioEdit (Hall 1999), ensuring data accuracy through manual correction. The Concatenate Sequence program in PhyloSuite (Zhang et al. 2020) was used to join the individual sequences, which were then saved in the fas. format. DNASP (Rozas 2009) was employed to calculate nucleotide diversity (π) and haplotype diversity (*Hd*), with the haplotype statistics for the *R. roxburghii* populations saved in the .csv format. The Median‐joining method in Network (Bandelt et al. 1999) was utilized to construct haplotype networks, and ArcGIS (ESRI, Redlands, CA, USA) was used in conjunction with sample collection information to plot the geographical distribution of haplotypes. Utilizing Permut (Pons and Petit 1996) to calculate total genetic diversity (*Ht*), within‐population genetic diversity (*Hs*), and the genetic differentiation coefficients *N*st and *G*st. If *N*st > *G*st, it suggests that the species has a significant phylogeographic structure, meaning that populations with similar haplotype genetic distances are associated with geographical proximity. If *N*st < *G*st, it indicates that the species lacks phylogeographic structure (Pons and Petit 1996). Arlequin (Excoffier and Lischer 2010) is employed for conducting AMOVA analysis and calculating the genetic differentiation coefficient (*F*st). Gene flow (*Nm*) was determined using the formula *Nm* = (1 − *Fst*)/4*Fst*. DNASP (Rozas 2009) was utilized to compute Tajima's D (Tajima 1983) and Fu and Li's D neutrality test values and to perform a mismatch analysis. This was done to assess whether the results conformed to a neutral evolutionary model and, in conjunction with the mismatch analysis, to determine whether the species had undergone expansion during its historical evolutionary process.

### 2.4 Modeling of Potential Habitats

A total of 464 distribution records were sourced from the Global Biodiversity Information Facility (GBIF) (http://www.gbif.org/) and field survey records. After removing duplicates and selecting natural occurrences, 157 valid distribution points were identified. Spatial resolution climate data at 2.5 arc‐minutes were retrieved from the WorldClim (http://worldclim.org) database for the Last Glacial Maximum (LGM; 22 ka BP), the mid‐Holocene (MH; 6 ka BP), the contemporary period (1950–2000), and the future (2070). The climate data for the LGM, MH, and various 2070s scenarios were produced using the CCSM4 general circulation model (Gent et al. 2011). The future climate data were selected based on a greenhouse gas emission scenario with a radiative forcing of 2.6 W/m^2^, corresponding to the Representative Concentration Pathway (RCP) 2.6. The raw data for each time period encompassed 19 bioclimatic variables. Utilizing DIVA‐GIS, values for these 19 climatic variables were extracted at 157 valid distribution points. Subsequently, SPSS Statistics software was employed to conduct Pearson correlation and principal component analysis to identify and exclude climate variables with high intercorrelations and low predictive contributions to the model accuracy. The MaxEnt (Phillips and Dudík 2008; Phillips et al. 2006) was utilized to predict the potential geographical distribution of *R. roxburghii* across the four periods based on these climate variables. The accuracy of the MaxEnt model predictions was assessed using the area under the receiver operating characteristic curve (AUC). Finally, the predicted results were imported into ArcGIS for suitability classification into four categories: unsuitable (0–0.1), low suitability (0.1–0.3), moderate suitability (0.3–0.6), and high suitability (> 0.6).

## 3 Results

### 3.1 ISSR‐PCR Amplification Results and Haplotype Distribution

**FIGURE 1:**
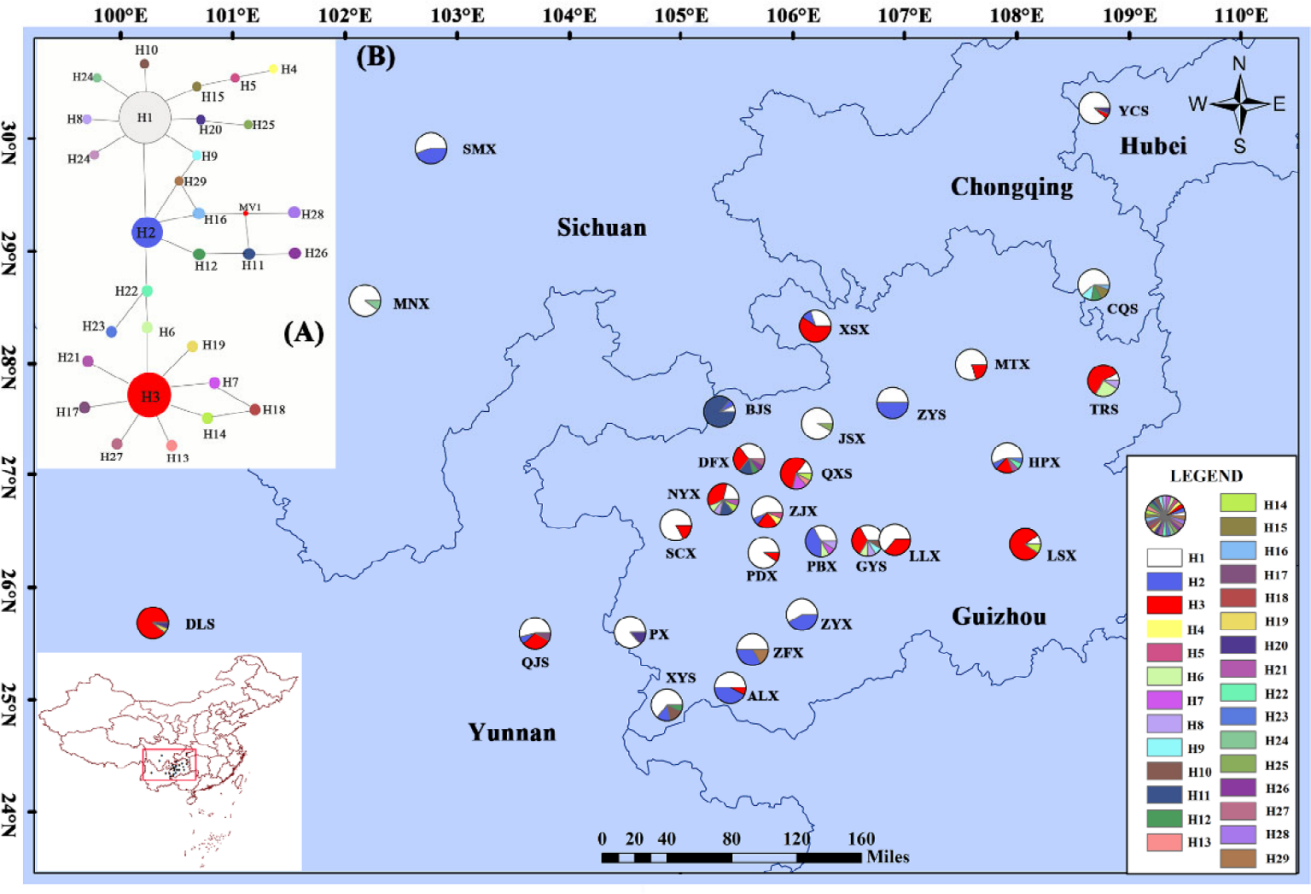
Haplotype mesh map (A) and haplotype geographical distribution map (B) constructed based on *Rosa roxburghii* Tratt chloroplast DNA (cpDNA). H1–H29 represent haplotypes 1–29, respectively, and the pie chart indicates the frequency of haplotypes in each population.

**FIGURE 2:**
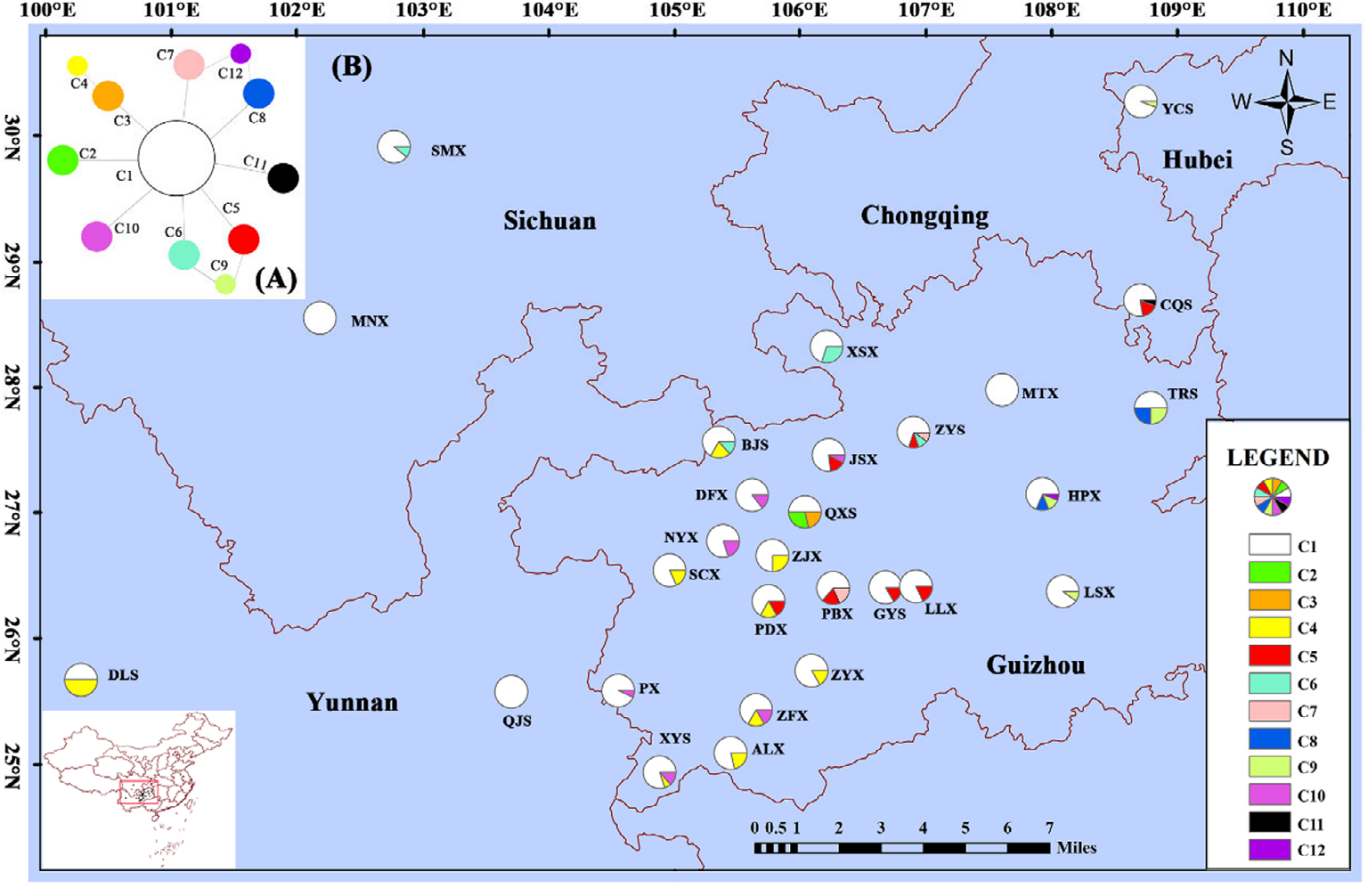
Haplotype mesh map (A) and haplotype geographical distribution map (B) constructed based on *R. roxburghii* single‐copy nuclear gene (SCNG), C1–C12 represent haplotypes 1–12, respectively, and the pie chart indicates the frequency of haplotypes in each population. Our present results are basically consistent with the previous structure. C1 accounts for the highest proportion in 28 populations (increasing ZYS population), and so does our previous research (R‐1) based on 27 populations (Wu et al. 2023).

We employed seven ISSR primers for the amplification of *R. roxburghii* , revealing a total of 79 loci detected at the species level, with 79 polymorphic bands and a polymorphism percentage reaching 100%, which indicates abundant genetic variation. Specifically, the loci 827 and 851 produced the least number of bands, whereas loci 834 and 835 generated the highest number of bands, suggesting the highest level of polymorphism (Table S1). Five cpDNA fragments were concatenated to form a combined sequence *psb*F‐*trn*H + *atp*F‐*trn*H + *trn*L‐*trn*F + *acc*D‐*psa*I + *trn*G‐*trn*S, with a total length of 3270 bp, including 16 variable sites and 11 parsimony informative sites, and a GC content of 29.37%. The details of each fragment are as follows: *psb*A‐*trn*H is 336 bp long with a GC content of 26.18%, with four variable sites and three parsimony informative sites; *atp*F‐*trn*H is 647 bp long with a GC content of 31.16%, with two variable sites and one parsimony informative site; *trn*L‐*trn*F is 585 bp long with a GC content of 21.54%, with three variable sites and two parsimony informative sites; *acc*D‐*psa*I is 427 bp long with a GC content of 31.68%, with three variable sites and two parsimony informative sites; *trn*G‐*trn*S is 1275 bp long with a GC content of 29.38%, with four variable sites and three parsimony informative sites. Concatenated sequences revealed 29 haplotypes (H1–H29) (Figure 1B), with populations such as QXS, DFX, NYX, ZJX, PBX, GYS, CQS, and HPX exhibiting more than five haplotypes, and the NYX population displaying the highest diversity of haplotypes. Unique haplotypes were found in populations QXS, CQS, QJS, DLS, HPX, MNX, DFX, NYX, and ZFX. Haplotype H1 was the most frequent, representing the primary haplotype, followed by haplotype H3 (Table 1). Two SCNG fragments were concatenated to form a combined sequence *ncpGs* + *GAPDH*, with a total length of 1494 bp, the length of the fragment is increased by 200 bp on the basis of Wu et al. (2023), including eight variable sites and seven parsimony informative sites, and a GC content of 39.92%. The *ncpGS* sequence is 749 bp long with a G + C content of 38.36%, featuring four variable sites and three parsimony informative sites; the *GAPDH* sequence is 745 bp long with a G + C content of 41.48%, with four variable sites and four parsimony informative sites. A total of 12 haplotypes were identified (Figure 2B), with ZYS and HPX having the most haplotypes, MTX, QJS, and MNX each having only one haplotype, and CQS and HPX possessing unique haplotypes. Haplotype C1 was the most frequent (Table 1).

### 3.2 Population Genetic Diversity

Genetic diversity among 28 *R. roxburghii* populations was analyzed based on ISSR molecular markers (Table 2): the total genetic diversity (*Ht*) and within‐population genetic diversity (*Hs*) were 0.3588 and 0.2026, respectively. Seven ISSR primers amplified a total of 79 loci at the species level in *R. roxburghii* . The number of polymorphic loci across populations ranged from 29 to 62, with the percentage of polymorphism (PPB) varying from 36.71%–79.75%. Notably, the QXS population exhibited the highest number of polymorphic loci, with a PPB of 79.75%, which was a 43.04% difference from the MNX population, which had the lowest PPB. The observed number of alleles (*Ne*) across populations ranged from 1.2590 to 1.4475, Nei's genetic diversity (*H*) ranged from 0.1452 to 0.2655, and Shannon's information index (*I*) ranged from 0.2121 to 0.4004. All genetic diversity parameters were positively correlated with the percentage of polymorphism. At the species level, the observed number of alleles (*Ne*) was 1.6176, Nei's genetic diversity (*H*) was 0.3583, and Shannon's information index (*I*) was 0.5336. At the population level, populations within Guizhou Province such as QXS, DFX, NYX, PX, XYS, PDX, ZJX, LLX, and those outside the province like SMX, CQS, YCS exhibited higher *PPB*, *Na*, *Ne*, *H*, and *I* values, while populations within the province like ZYX, GYS, MTX, and those outside like MNX had relatively lower indices, with QXS showing the richest genetic diversity.

**TABLE 2:** Genetic diversity parameters of *R. roxburghii* populations based on ISSR, cpDNA, and SCNG.

Pop.	ISSR						cpDNA		SCNG	
	Polymorphic bits	PPB (%)	*Na*	*Ne*	*H*	*I*	*Hd*	*π*	*Hd*	*π*
QXS	62	79.75%	1.7975	1.4475	0.2655	0.4004	0.670	0.00049	0.440	0.00029
JSX	45	55.70%	1.5570	1.2817	0.1720	0.2643	0.154	0.00009	0.410	0.00029
DFX	54	67.09%	1.6709	1.3718	0.2176	0.3284	0.791	0.00080	0.264	0.00018
NYX	56	70.89%	1.7089	1.4084	0.2399	0.3609	0.867	0.00086	0.343	0.00023
SCX	50	56.96%	1.5696	1.3135	0.1864	0.2824	0.303	0.00037	0.439	0.00031
PX	48	60.76%	1.6076	1.3404	0.1990	0.2994	0.248	0.00008	0.133	0.00009
XYS	48	60.76%	1.6076	1.3368	0.2017	0.3058	0.638	0.00026	0.362	0.00026
ALX	47	59.49%	1.5949	1.3180	0.1894	0.2883	0.604	0.00030	0.363	0.00024
ZFX	43	54.43%	1.5443	1.3362	0.1947	0.2904	0.733	0.00029	0.600	0.00045
ZYX	40	48.10%	1.4810	1.2729	0.1607	0.2423	0.530	0.00016	0.303	0.00020
PDX	53	67.09%	1.6709	1.4474	0.2546	0.3745	0.182	0.00022	0.545	0.00041
ZJX	52	65.82%	1.6582	1.4074	0.2354	0.3491	0.659	0.00069	0.560	0.00042
PBX	45	56.96%	1.5696	1.3720	0.2078	0.3059	0.758	0.00070	0.652	0.00060
GYS	36	45.57%	1.4557	1.2841	0.1623	0.2409	0.818	0.00075	0.303	0.00020
LLX	48	60.76%	1.6076	1.4023	0.2298	0.3387	0.509	0.00062	0.327	0.00022
TRS	44	55.70%	1.5570	1.3215	0.1882	0.2828	0.636	0.00043	0.682	0.00055
XSX	43	54.43%	1.5443	1.3278	0.1927	0.2882	0.600	0.00063	0.467	0.00031
BJS	42	53.16%	1.5316	1.2988	0.1703	0.2548	0.242	0.00011	0.533	0.00040
ZYS	42	53.16%	1.5316	1.3824	0.2117	0.3079	0.556	0.00017	0.533	0.00048
MTX	31	39.24%	1.3924	1.2773	0.1540	0.2252	0.400	0.00049	0.000	0.00000
SMX	51	64.56%	1.6456	1.4175	0.2352	0.3466	0.523	0.00016	0.209	0.00014
CQS	54	68.35%	1.6835	1.4240	0.2452	0.3632	0.621	0.00033	0.386	0.00027
QJS	47	59.49%	1.5949	1.4006	0.2283	0.3356	0.654	0.00062	0.000	0.00000
DLS	44	55.70%	1.5570	1.3170	0.1885	0.2838	0.216	0.00010	0.529	0.00035
LSX	45	56.96%	1.5696	1.2749	0.1683	0.2603	0.378	0.00031	0.200	0.00013
YCS	51	64.56%	1.6456	1.4221	0.2433	0.3599	0.228	0.00018	0.118	0.00008
HPX	44	55.70%	1.5570	1.3139	0.1855	0.2806	0.675	0.00061	0.525	0.00044
MNX	29	36.71%	1.3671	1.2590	0.1452	0.2121	0.200	0.00012	0.000	0.00000
Species level	79	100%	2.0000	1.6176	0.3583	0.5336	0.692	0.00086	0.409	0.00031

Based on the analysis of chloroplast DNA (cpDNA) and single‐copy nuclear gene (SCNG) fragments, we found that the genetic diversity of cpDNA (*Ht* = 0.705, *Hs* = 0.483, *Hd* = 0.6921, *π* = 0.00086) was higher than that of SCNG (*Ht* = 0.253, *Hs* = 0.224, *Hd* = 0.4095, *π* = 0.00031) (Table 2). The NYX population exhibited the highest haplotype diversity (*Hd* = 0.867) and nucleotide diversity (*π* = 0.00086) in cpDNA, followed by GYS. Additionally, several populations located in the northwest (NYX, QXS, DFX) and northeast (CQS, TRS) of Guizhou Province had generally higher *Hd* and *π* values, consistent with the results based on ISSR studies. The TRS population showed the highest SCNG genetic diversity and nucleotide diversity (*Hd* = 0.682, *π* = 0.00055), with PBX being the next highest.

### 3.3 Population Genetic Differentiation

The results of genetic differentiation based on ISSR (Table 3) revealed a genetic differentiation coefficient *Gst* of 0.4353, which is greater than 0.25, and a gene flow *Nm* of 0.8515, which is less than 1. This indicates that 41% of the variation is between populations and 59% is within populations, suggesting that genetic differentiation in *R. roxburghii* is primarily within populations. The molecular variance analysis (AMOVA) based on cpDNA (Table 3) showed that the percentage of genetic variation within populations (59%) was higher than that between populations (41%). The genetic differentiation coefficient *Fst* was 0.406, significantly greater than 0.25, while *Gst* was 0.3150 and *Nst* was 0.3530 (*p* > 0.05), with *Nm* calculated as 0.7400. The AMOVA results based on SCNG (Table 3) indicated that the percentage of genetic variation within populations (86%) was significantly higher than that between populations (14%). The genetic differentiation coefficient *Fst* ranged from 0.05 to 0.15, with *Gst* at 0.1150 and *Nst* at 0.1350 (*p* > 0.05), and *Nm* was 1.8000.

**TABLE 3:** Molecular analysis of variance (AMOVA) based on ISSR, cpDNA, and SCNG.

Source of variation	D.f.	Sum of squares	Variance components	Percentage of variation	Differentiation coefficient
**ISSR**					
Among populations	27	2296.757	85.065	41.000	0.435
Within populations	336	2899.946	8.631	59.000	
Total	363	5196.703	14.523		
**cpDNA**					
Among populations	27	171.671	0.442	40.593	0.406
Within populations	336	216.494	0.646	59.407	
Total	363	388.165	1.088		
**SCNG**					
Among populations	27	21.634	0.042	13.642	0.136
Within populations	336	88.195	0.263	86.358	
Total	363	109.829	0.305		

### 3.4 Population Genetic Structure and Relationships

**FIGURE 3:**
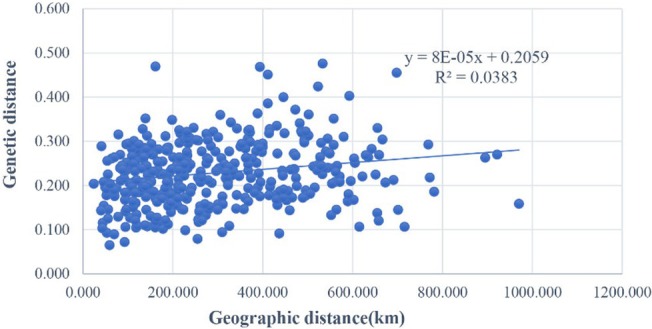
Mental detection based on genetic distance and geographical distance of *R. roxburghii* populations.

We explored the relationship between genetic distance and genetic concordance among *R. roxburghii* populations (Table S2). The results indicated that the genetic distance ranged from 0.0653 to 0.4762, with the smallest genetic distance (0.0653) and highest genetic concordance (0.9367) observed between the ALX and XYS populations within Guizhou Province. A Mantel correlation test was conducted using the actual geographical distances and genetic distances of *R. roxburghii* populations (Figure 3), resulting in a regression equation of y = 8E‐05*x* + 0.2059, *R* ^*2*^ = 0.0383, *p* = 0.024 < 0.05. The results indicate that there is a significant positive correlation between the genetic distance and geographical distance of *R. roxburghii* populations. However, the geographical distance only accounts for 3.83% of the variation in genetic distance, suggesting a very weak correlation between the two.

Based on the genetic distance matrix calculated from ISSR molecular markers, a phylogenetic tree was constructed using the neighbor‐joining (NJ) method (Figure S3). The results indicated that the populations MNX and BJS each formed a separate branch, while the remaining populations clustered into a single major branch. Within this branch, populations ZJX, PDX, SMX, CQS, PBX, LLX, XSX, GYS, TRS, and MTX formed one sub‐branch, and QXS, JSX, DFX, SCX, XYS, ALX, and QJS formed another sub‐branch, the other populations of PX, ZFX, LSX, YCX, ZYX, NYX, ZYS, DLS, and HPX are also included in this sub‐branch. The network diagrams constructed based on cpDNA haplotypes (Figure 1A) and SCNG haplotypes (Figure 2A) revealed that in the cpDNA haplotypes, other haplotypes were derived with H1, H2, and H3 as the central haplotypes, resulting in a star‐like distribution of the entire haplotype network. Combined with the geographical distribution of haplotypes (Figure 1B), H1 and H3 were the most frequent, followed by H2, suggesting that they are ancient haplotypes. In the SCNG haplotypes, other haplotypes were derived with C1 as the central haplotype, and C1 had the highest proportion in all populations (Figure 2A,B), suggesting that it is an ancient haplotype.

Using the closely related species *Rosa cymosa* Tratt. as an outgroup, phylogenetic trees for cpDNA (Figure S4A) and SCNG (Figure S4B) haplotypes were constructed based on the maximum likelihood (ML) method. The cpDNA haplotypes are distinctly divided into three branches. Clade A, with H1 as the central haplotype, forms clusters of H9, H29, H15, H4, H5, H8, H21, H20, H25, H10, and H24. Clade B is centered around H2, including H16, H28, H12, H11, H27, H17, and H26. Clade C comprises H22, H23, H6, H19, H7, H14, H18, H13, with H3 as the central haplotype. These results were consistent with the haplotype network diagrams. The phylogenetic tree based on SCNG haplotypes also showed a close correspondence with the haplotype network diagrams.

### 3.5 Population History and Range Dynamics

**FIGURE 4:**
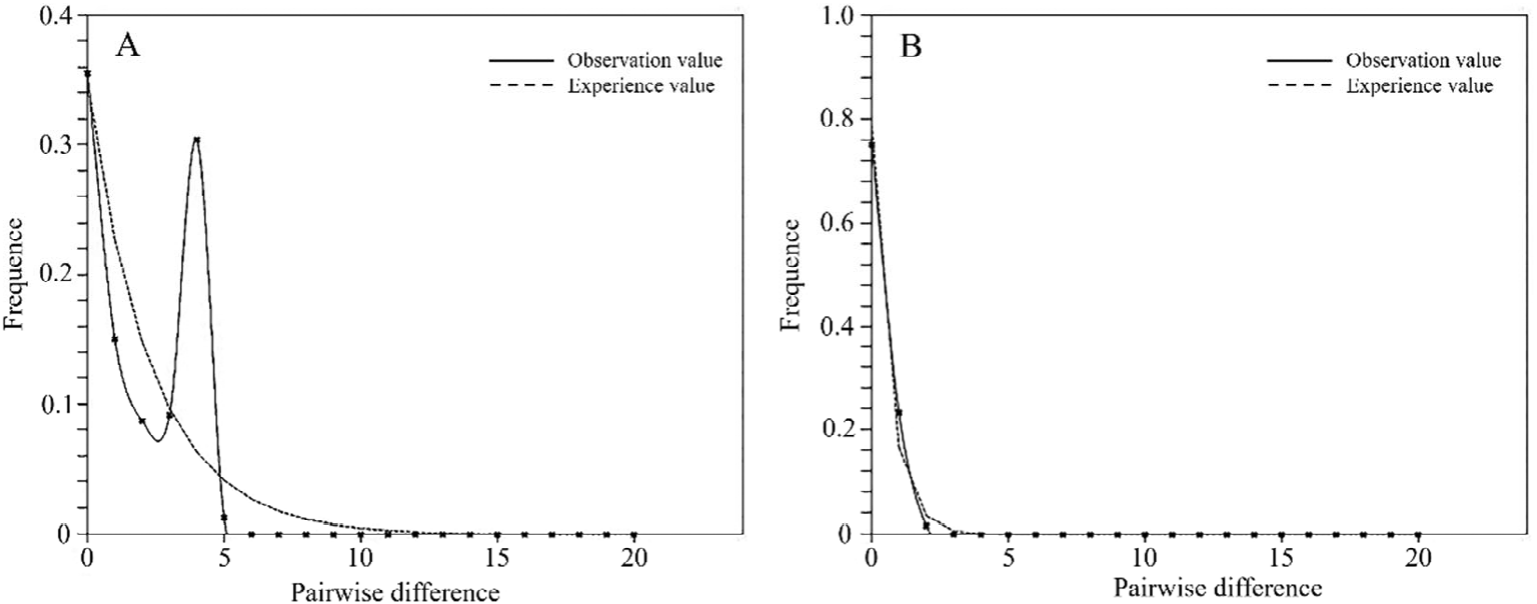
Mismatch analysis plot for the chloroplast genes (A) and single‐copy nuclear genes (B) of *R. roxburghii*.

Neutrality test results showed that the Tajima's D statistic for *R. roxburghii* cpDNA was −0.41979 (*p* > 0.10), Fu and Li's D* value was −1.60206 (*p* > 0.10), and Fu and Li's F* value was −1.37386 (*p* > 0.10), all of which were consistent with the neutral evolution model and did not deviate from neutral equilibrium. Similarly, for SCNG, the Tajima's D statistic was −1.29257 (*p* > 0.10), Fu and Li's D* value was −0.22501 (*p* > 0.10), and Fu and Li's F* value was −0.38866 (*p* > 0.10), all aligning with the neutral evolution model and not deviating from neutral equilibrium. The mismatch analysis results for cpDNA (Figure 4A) and SCNG (Figure 4B) revealed a bimodal distribution for cpDNA and a unimodal distribution for SCNG. This suggests that *R. roxburghii* may have undergone a recent small‐scale population expansion event without deviating from neutral equilibrium.

**FIGURE 5:**
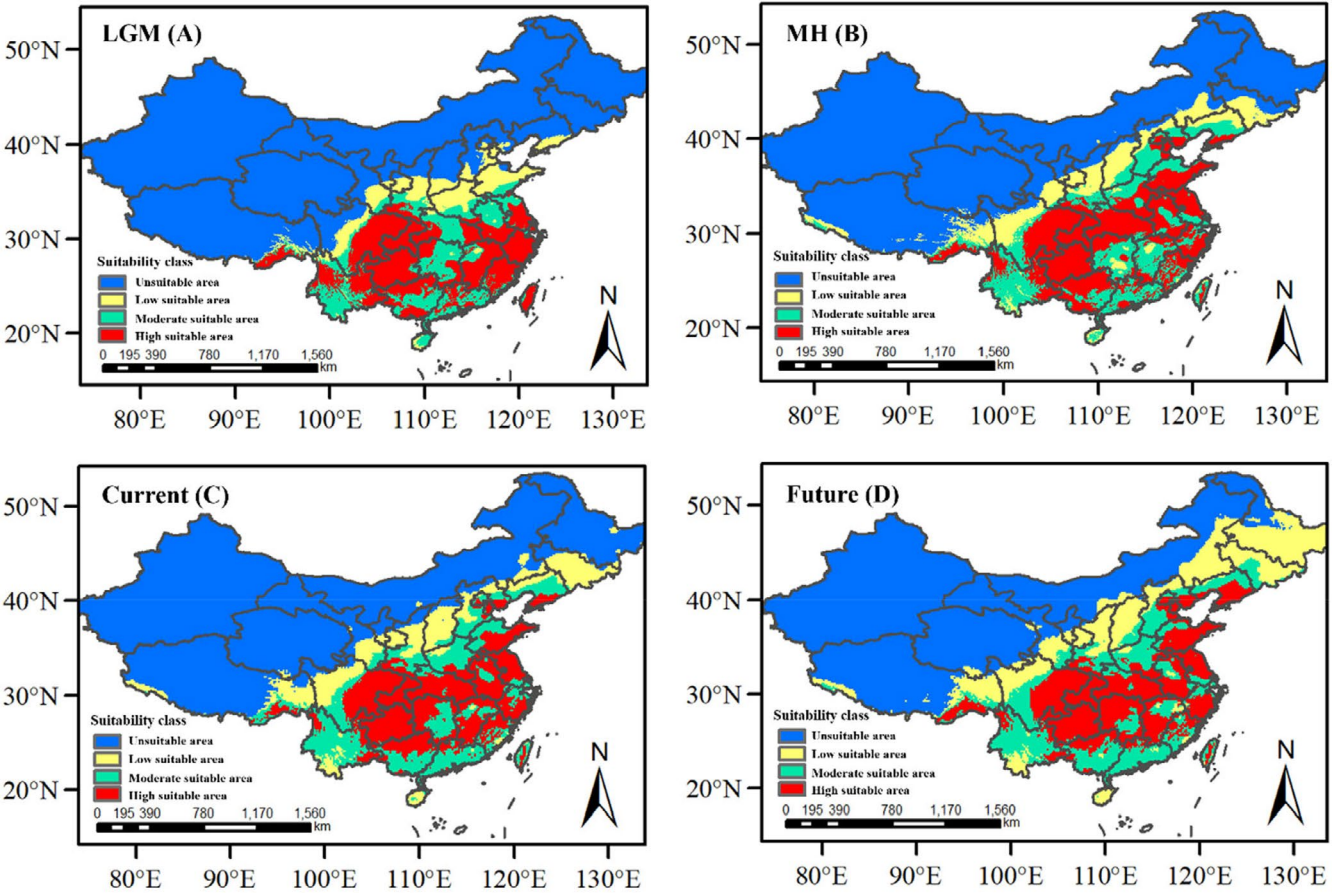
Potential distribution of *R. roxburghii* based on the MaxEnt model during the last glacial maximum (LGM) (A), the middle holocene (MH) (B), the current (C), and the future (D). Red: High suitable area; Yellow: Moderate suitable area; Green: Low suitable area; Blue: Unsuitable area.

Using the MaxEnt model, we simulated and predicted the geographical distribution patterns of *R. roxburghii* during the last glacial maximum (LGM), mid‐Holocene, present day, and the year 2070. The results indicated that the climate factors with the highest contribution rates across all periods were primarily related to temperature, including the mean monthly range of diurnal temperature (bio2), temperature seasonality (bio4), minimum temperature of the coldest month (bio6), mean temperature of the driest quarter (bio9), mean temperature of the coldest quarter (bio11), precipitation variability (bio15), and precipitation of the warmest quarter (bio18). The average AUC value was 0.991, demonstrating high stability and accuracy of the model. The potential high suitability areas for *R. roxburghii* were mainly concentrated in the southwestern regions of Guizhou, Chongqing, and Sichuan provinces, as well as in Yunnan, Guangxi, Hubei, Hunan provinces, and the eastern regions of China. The predicted core distribution areas were largely consistent with the current distribution points. Historical changes in the potential distribution areas of *R. roxburghii* over the four periods (Figure 5) showed that from the LGM (Figure 5A) to the mid‐Holocene (Figure 5B), there was an increase in the total suitable area, with an expansion of the moderately and highly suitable areas, and a gradual spread of suitable regions towards the higher latitudes in the east. Comparing the species distribution during the mid‐Holocene (Figure 5B) and the present day (Figure 5C), the core distribution area of *R. roxburghii* remained unchanged, while the range of some highly and moderately suitable areas decreased. Comparing the present day (Figure 5C) and the year 2070 (Figure 5D), the total suitable distribution area is predicted to increase compared with the present day, with a trend of migration towards higher latitude regions.

## 4 Discussion

### 4.1 Genetic Diversity of *R. roxburghii* Populations

This study, based on data and observations from ISSR, cpDNA, and SCNG molecular markers, supports the presence of high genetic diversity in *R. roxburghii* , with varying degrees of variation among populations. Research using ETS‐SSR on 12 wild *R. roxburghii* populations in Guizhou Province, China, also indicated that *R. roxburghii* exhibits a moderate to high level of genetic diversity (Zhang et al. 2017). In the study of the genetic relationship of the genus *Rosa L*., Suprun et al. (2020) utilized five ISSR markers, indicating that the number of polymorphic alleles ranged from 12 to 31 fragments. Studies using ISSR on plants of the genus *Crataegus* in the Rosaceae family revealed that *Crataegus songorica* K. Koch. has high genetic diversity (*PPB* = 98.35%, *Ne* = 1.5523, *H* = 0.3268, *I* = 0.4939) (Sheng 2017). The results of this study, based on ISSR molecular markers, show that *R. roxburghii* has a polymorphism rate of 100% at the species level. The effective number of alleles (*Ne*) is 1.6176, and Nei's genetic diversity index (*H*) is 0.3583, with the Shannon information index (*I*) at 0.5336, indicating high genetic diversity in *R. roxburghii* . There is considerable genetic diversity among *R. roxburghii* populations, with each population's genetic diversity level slightly lower than that at the species level. At the population level, QXS exhibits the highest genetic diversity (*PPB* = 79.75%, *Na* = 1.7975, *Ne* = 1.4475, *H* = 0.2655, *I* = 0.4004). Furthermore, populations in the northwest (QXS, DFX, NYX), northeast (CQS), and southwest (PX, XYS) directions of Guizhou Province all have higher genetic diversity than populations in other regions. Based on cpDNA genetic diversity analysis, the total haplotype diversity (*Hd*) of *R. roxburghii* at the species level is 0.692, and the total nucleotide diversity (*π*) is 0.00086. This exceeds the values reported for other Rosaceae species such as wild *Prunus pseudocerasus* (*Hd* = 0.5620, *π* = 0.0018) (Chen et al. 2012); cultivated *Prunus pseudocerasus* (*Hd* = 0.5590, *π* = 0.001) (He et al. 2014); and *Cerasus serrulata* (*Hd* = 0.5530, *π* = 0.0014) (Peng 2021) in recent years, further indicating that the *R. roxburghii* species possesses high genetic diversity. At the population level, it was observed that populations such as QXS, DFX, NYX, CQS, TRS, GYS, and PBX exhibit higher genetic diversity, which is consistent with the results of ISSR molecular marker analysis, especially when highlighting the genetic diversity in the northwest (QXS, DFX, NYX) and northeast (CQS) of Guizhou Province. This consistency indicates that different molecular marker methods can corroborate each other and strengthen the assessment of population genetic diversity. In comparison to cpDNA, the genetic diversity analysis of *R. roxburghii* 's single‐copy nuclear genes reveals lower genetic diversity, which is in agreement with previous studies (Nuer et al. 2015) on *Prunus armeniaca* L. in the Rosaceae family using GAPDH, and similar to the findings of Wright et al. (2005) and Kilian et al. (2006). However, the genetic diversity of most populations based on both cpDNA and SCNG molecular markers is similar, such as populations TRS and PBX, suggesting that both markers can corroborate each other and provide additional genetic information.

The high genetic diversity of *R. roxburghii* may be attributed to factors such as its geographical distribution, life habits, and reproductive system (including flowering, pollination, fertilization, development, self‐compatibility, and mating systems) (Hamrick et al. 1992). Since *R. roxburghii* is primarily distributed in the southwestern region of China, which has a subtropical humid monsoon climate characterized by warm and moist conditions, the topography is predominantly mountainous and hilly with complex mountain ranges, creating favorable conditions for the growth of *R. roxburghii* (Chi et al. 2012; Ding 2015). *R. roxburghii* thrives in warm and humid climates (Fan and Gong 2019), exhibits strong vitality, and can tolerate certain low temperatures, mainly found along roadsides, field edges, water ditches, and wastelands (Zhang 2015). Different ecological environmental factors (soil, water, temperature, light) lead to different phenotypes in populations as they adapt to their surroundings, amplifying genetic variation among different populations from the outside in (Liu 2020). Most *Rosa* plants can undergo both self‐pollination and cross‐pollination, and there is a tendency for species populations to hybridize naturally through insect and wind vectors, with a certain rate of outcrossing within and between populations, and even between different genera (Deng 2015). Moreover, the *R. roxburghii* used in the study is found in biodiversity hotspots, which may experience small‐scale long‐distance dispersal due to human collection activities, predation by birds and animals, and water flow. These factors contribute to the high genetic diversity of *R. roxburghii* .

### 4.2 Genetic Structure of *R. roxburghii* Populations

Genetic structure describes the distribution patterns of genetic diversity and variation within and among different populations of a species over time and space, including genetic variation within populations and genetic differentiation between populations. This is often influenced by factors such as gene flow, genetic drift, natural selection, geographic isolation, migration, and diffusion (Wang et al. 2005; Wang 2016a, 2016b). Our AMOVA analysis based on ISSR data revealed that genetic differentiation in *R. roxburghii* is primarily within populations (59%), but there is also significant genetic differentiation between populations, consistent with the findings of Ma et al. (2013) and He (2006) on Rosaceae plants. The genetic differentiation coefficient (*Gst*) for populations was 0.435, greater than 0.25, and the gene flow (*Nm*) was 0.8515, less than 1, indicating a significant degree of genetic differentiation in *R. roxburghii* populations with some degree of gene exchange. Based on the genetic distance and genetic concordance derived from ISSR, we found that the populations ALX and XYS, which are geographically close in Guizhou Province, had the smallest genetic distance (0.0653) and the highest genetic concordance (0.9367), suggesting that populations with smaller genetic distances have higher genetic consistency. The Mantel correlation test results indicated that there is a significant positive correlation between geographical distance and genetic distance (*R* ^*2*^ = 0.0383, *p* = 0.024 < 0.05), suggesting that while geographical distance is an important factor affecting genetic distance, it is not the primary factor, and genetic drift may be the dominant factor driving genetic variation within populations. The NJ phylogenetic tree constructed from the genetic distance matrix shows that the MNX and BJS populations each form a separate branch, while the remaining populations cluster into a larger group. One possible explanation is that some degree of gene flow among populations has led to populations that are geographically close, such as QXS, JSX, DFX, SCX, XYS, ALX, PDX, and ZJX, clustering into a single branch. In contrast, the MTX and MNX populations are separated by a greater distance, and the complex terrain, including numerous rivers and mountain ranges, between the two locations acts as a natural barrier to gene flow. Another explanation is that populations such as MNX and BJS may have originated from ancient natural groups.

Through the study of chloroplast genes (*Ht* = 0.7050, *Hs* = 0.4830, *Fst* = 0.4059 > 0.25, *Nst* = 0.3530 > *Gst* = 0.3150 (*p* > 0.05), *Nm* = 0.7400), it was found that there is a high level of genetic differentiation among *R. roxburghii* populations without a significant phylogeographic structure. This is consistent with the results of a study on *Rubus foliolosus* D. Don species in the Rosaceae family using two chloroplast DNA combined fragments (*Hs* = 0.689; *Ht* = 0.768; *Gst* = 0.1030; *Nst* = 0.1330, *Nm* = 1.8000) (Zhang 2017), but differs from the genetic diversity analysis of *R. roxburghii* based on two chloroplast fragments (*Gst* = 0.1360, *Nst* = 0.2568; *Nst* significantly greater than *Gst*) (Zhang et al. 2017). The study of single‐copy nuclear genes (*Ht* = 0.2530, *Hs* = 0.2240, *Fst* = 0.1360, 0.05 < *Fst* < 0.15, *Gst* = 0.1150 < *Nst* = 0.1350 [*p* > 0.05]) also indicates that there are a large number of closely related haplotypes among different populations of *R. roxburghii* , with no apparent phylogeographic structure. Species with a high proportion of unique haplotypes often do not reveal a clear phylogeographic pattern (Geng et al. 2018). In our study, most haplotypes are unique to each population, and even a single population may fix multiple unique haplotypes, indicating that there is discernible genetic variation within some *R. roxburghii* populations. The haplotype geographical distribution map also shows that the interweaving distribution of haplotypes within populations is not significantly correlated with their geographical distribution, further confirming the absence of a clear phylogeographic structure in *R. roxburghii* .

The AMOVA results indicated that the percentage of genetic variation within *R. roxburghii* populations (59%), based on cpDNA, was higher than that between populations (41%). This suggests that the genetic variation in *R. roxburghii* is primarily occurring within populations. However, the difference in genetic variation between and within populations was not significant, indicating that there is also a degree of genetic variation between populations. The results based on SCNG sequences showed that the genetic variation within populations (86%) was significantly higher than that between populations (14%), which aligns with previous findings on Prunus *mahaleb* , a Rosaceae plant (81.8% within‐population genetic variation, 16.5% between‐population genetic variation) (Jordano and Godoy 2000). They attributed the high within‐population genetic variation to the significant influence of factors such as geography, altitude, and nonoverlapping flowering and fruiting periods. Species that possess both self‐fertilization and outcrossing reproductive systems often exhibit high genetic differentiation (Nybom 2004; Wright 1951, 1965). The high genetic differentiation in *R. roxburghii* may be due to its reproductive system causing changes in the patterns of genetic variation among populations. Another explanation is that the environmental heterogeneity, geographic isolation, and gene flow in the southwestern region may exert different selective pressures among populations, leading to the emergence of genetic differentiation.

### 4.3 Comparative Patterns of cpDNA and SCNG Markers

The genetic differentiation level (*Fst* = 0.4059) based on cpDNA markers in *R. roxburghii* populations is higher than that of SCNG (*Fst* = 0.1364). Nuclear gene molecular markers are primarily dispersed through two modes of gene flow (pollen flow and seed flow); in contrast, chloroplast genes, which are uniparentally inherited and do not undergo genetic recombination during inheritance, are mainly dispersed through seed flow (Petit et al. 2005). In this study, a discrepancy in genetic differentiation was observed between cpDNA sequence‐based and SCNG sequence‐based studies. The smaller genetic differentiation between populations based on SCNG may be due to the fact that nuclear genomic molecular markers, which have both paternal and maternal inheritance, tend to reduce the level of genetic differentiation between populations (Rendell and Ennos 2002). Therefore, the greater genetic structure observed in chloroplast data likely indicates that gene flow through pollen in *R. roxburghii* is higher than through seeds. Indeed, the calculated effective gene flow for both marker types also shows that gene flow through pollen (*Nm* = 1.8000) is twice that of seeds (*Nm* = 0.7400). In this system, the limited movement of seeds between populations could be due to several factors: Firstly, several neighboring populations within the same geographical area do not share haplotypes, indicating that even short‐distance seed dispersal is limited, which explains the unique haplotypes found in populations in adjacent areas of the southwestern region. Secondly, the weight of *R. roxburghii* fruits typically restricts seed dispersal to the vicinity of the plant without external forces, and human activities (construction of roads, urban expansion, dam building, and land development) have led to fragmented distribution of *R. roxburghii* populations. Additionally, limited bird and animal predation dispersal and the barriers of plateau mountains have hindered long‐distance seed flow between populations.

### 4.4 Glacial Refugia and Potential Optimal Distribution Areas

According to the theory of retrogenesis in phylogeography, haplotypes located at the center of a haplotype network are considered ancient (Avise et al. 1987; Hu et al. 2019). Notably, populations with high genetic diversity (haplotype diversity and nucleotide diversity) and those possessing unique haplotypes may represent the center of origin or glacial refugia for the species during the Quaternary ice ages (Hewitt 1996). Populations in glacial refugia often need to maintain higher genetic diversity and unique haplotypes than populations formed by migration or dispersal to avoid reducing haplotype polymorphism due to genetic drift, founder effects, and other events during migration or dispersal (Hewitt 2000). Based on cpDNA and SCNG haplotype studies, it is inferred that H1, H2, H3, and C1 are ancestral haplotypes. Combining the genetic diversity results from ISSR, cpDNA, and SCNG, it is suggested that the NYX, DFX, and QXS populations in the northwest of Guizhou and the HPX, PBX, and GYS populations in central Guizhou have high genetic diversity, a greater number of haplotype types, ancient haplotypes, and unique haplotypes. Therefore, it is hypothesized that the northwest and central regions of Guizhou may have been the distribution center and glacial refugia for *R. roxburghii* in the southwestern region of China during the Quaternary ice ages. The TRS and CQS populations, located in the Wuling Mountains, both have high genetic diversity, with CQS possessing the unique haplotype C11. In addition, considering different molecular markers, it is speculated that there were two or more glacial refugia for *R. roxburghii* in the southwestern region during the Quaternary ice age. Some phylogeographic studies have also reported (Yan 2010) that the southwestern region south of the Qinling Mountains served as a natural barrier that effectively mitigated the impact of glacial spread on the area, with mountain ranges like the Daloushan and Wuling Mountains, as well as basins and north–south oriented river valleys, providing favorable conditions for species protection and migration. The results predicted by the MaxEnt model indicate that the high suitability areas for *R. roxburghii* are located in Guizhou Province and Chongqing City, further supporting the conclusion that *R. roxburghii* may have had multiple glacial refugia during the ice age.

Neutrality tests combined with mismatch analysis suggest that *R. roxburghii* may have undergone small‐scale population expansion events without deviating from neutral equilibrium. This could be associated with the existence of multiple glacial refugia and postglacial expansion for *R. roxburghii* populations (Qiu et al. 2011; López‐Pujol et al. 2011). On one hand, it is possible that several interglacial periods occurred during the Quaternary ice age, with warm and humid climates that were conducive to plant growth and expansion (Wang 2016a, 2016b). Consequently, *R. roxburghii* may have migrated and expanded from Guizhou Province to surrounding areas after the Quaternary ice age. On the other hand, due to the barrier of mountain ranges in the southwestern region, geographic isolation led to *R. roxburghii* undergoing slow, short‐distance migration, resulting in the current haplotype geographical distribution pattern. Overall, whether based on SCNG or cpDNA, the haplotype types, haplotype diversity, and nucleotide diversity outside of Guizhou Province are lower than those within Guizhou. Combined with the inference of glacial refugia and expansion routes, it is suggested that Guizhou, located in the southwestern region, is the center of origin and evolution for *R. roxburghii* , with *R. roxburghii* populations in other provinces likely originating from Guizhou. This is consistent with the findings of Zhang (2017).

This study has revealed the key climatic factors influencing the distribution of *R. roxburghii* , which are primarily temperature‐related, reflecting the species' sensitivity to temperature changes. During the last glacial maximum (LGM), under the backdrop of global cooling, *R. roxburghii* retreated toward the equator and shifted southward. In the mid‐Holocene (MH), the potential distribution area of *R. roxburghii* expanded significantly, migrating eastward to reach the coastal regions of East China. This is consistent with Wang Wencai's distribution pattern of East Asian flora, characterized by a distribution from southwest to east and a migration route from the southwestern part of China directly to the coastal areas of East China (Wang 1992). This change may be associated with global warming and glacial retreat, providing *R. roxburghii* with a broader range of suitable habitats. In the modern era, *R. roxburghii* is found in areas with high distribution fragmentation, and the highly suitable and moderately suitable areas have significantly contracted, which may be related to human activities and climate change. Predictions from the present to the year 2070 indicate that the total suitable distribution area for *R. roxburghii* will further increase, with a trend of migration towards higher latitudes, potentially indicating the future impact of global warming on the distribution of *R. roxburghii* . The core distribution area of *R. roxburghii* is mainly in the southwestern region, consistent with previous research results (Fan et al. 2021b). Existing data also suggest that climate warming leads to species migrating toward higher latitudes and altitudes (Kelly and Goulden 2008; Ma et al. 2015). Clearly, the distribution range of a species is largely influenced by climate and is a key factor determining species distribution on a large scale.

### 4.5 Conservation Insights

Genetic diversity is closely related to the evolutionary potential and reproductive adaptability of populations, and maintaining genetic diversity is a primary focus in the management of wild populations (Wang et al. 2022). *R. roxburghii* is a highly valuable ornamental fruit with both medicinal and edible qualities. It is renowned for its high content of vitamin C, superoxide dismutase, and flavonoids, and is acclaimed as the “King of Vitamin C” (Fan et al. 2021a). Based on it is rich in nutrients, safe for consumption, and traditionally used in Chinese food and medicine, *R. roxburghii* products have been extensively applied in the food, pharmaceutical, health product, and daily chemical industries, with broad market prospects. However, due to continuous harvesting, habitat destruction, and fragmentation, the number of wild populations has declined. Therefore, proposing a priority conservation strategy for wild *R. roxburghii* germplasm resources is imperative. It is worth noting that maintaining an effective population size of germplasm should be a priority in the conservation plan (Hu et al. 2023). This study indicates that several populations in the northwest direction of Guizhou Province (DFX, NYX, QXS, and BJS) and the central Guizhou direction (HPX, PBX, and GYS) have relatively high genetic diversity and are refugial populations, while The TRS and CQS populations, located in the Wuling Mountains, both possess high genetic diversity and unique haplotypes. The high suitability areas for *R. roxburghii* are in regions such as Guizhou Province and Chongqing City, which can be considered key areas for restoring wild populations. Sichuan Province, Yunnan Province, Guangxi Province, Hubei Province, and Hunan Province can be considered as priority areas for expanding the distribution of *R. roxburghii* . Therefore, the populations of above areas should be given priority conservation. What is more, due to geographical isolation, the population of different areas, which should be conserved as an independent unite. We can carry out in situ conservation strategies. On the other hand, for these five priority areas, tissue culture (Kasim et al. 2021; Onay et al. 2011) can be used to solve the problems of plant regeneration commendably. When the successful and well‐regenerated plants were obtained, through artificial cultivation methods can reduce inbreeding in *R. roxburghii* and protect the genetic diversity of existing wild populations. Based on in situ conservation, we can collect the seeds of individuals in the unique haplotype population and smaller population sizes (Hu et al. 2023; Wu et al. 2024). Totally, there are four suggestions: (1) carry out in situ conservation strategies, It is well known that this is an effective and convenient protection strategy; (2) implement ex situ conservation, we can collect the seeds of *R. roxburghii* and establish a germplasm resource bank, it is good for protecting the germplasm resource of *R. roxburghii* ; (3) use tissue culture rationally, it is effective to maintain the genetic diversity of *R. roxburghii* ; (4) call on local villagers to participate in conservation efforts and avoid indiscriminate logging. The population genetic pattern information obtained from the hotspot areas in this study has positive implications for the conservation of wild resources and genetic improvement.

## 5 Conclusions

This study comprehensively examined the genetic diversity, genetic differentiation, phylogeographic structure, population historical dynamics, and potential optimal distribution areas of *R. roxburghii* by analyzing a total of 364 specimens from 28 populations in the southwestern region of China using ISSR, cpDNA, and single‐copy nuclear gene data, in conjunction with the MaxEnt model. The results revealed that the origin and distribution center of *R. roxburghii* are located in Guizhou Province, with populations in other provinces likely having originated from Guizhou. During the Quaternary ice age, *R. roxburghii* had at least two glacial refugia in the region and experienced a small‐scale population expansion. The distribution pattern of *R. roxburghii* underwent changes of expansion, contraction, and subsequent expansion across four periods. Climate fluctuations and geographic isolation were the main factors affecting the genetic differentiation of *R. roxburghii* . By integrating molecular systematic geography, phylogenetic methods, and species distribution models, this study not only provides a better understanding of the genetic background of *R. roxburghii* , offering a scientific basis for its conservation and sustainable utilization, but also presents a new case study for understanding the biodiversity of the southwestern region of China.

## Author Contributions

**Cai Zhao:** formal analysis (equal), investigation (equal), methodology (equal), software (equal), supervision (equal), writing – review and editing (equal). **Shanshan He:** methodology (supporting), software (equal), writing – original draft (equal). **Feng Pan:** investigation (equal), methodology (equal), resources (equal). **Chunxue Jiang:** software (equal). **Jian Feng:** data curation (equal), formal analysis (equal). **Jian Jian Wang:** supervision (supporting).

## Conflicts of Interest

The authors declare no conflicts of interest.

## Supporting information


Appendix S1.

## Data Availability

All haplotype sequences in this study are openly available in Genbank at https://www.ncbi.nlm.nih.gov/genbank/, reference number OP654768–OP654795, OP750062–OP750177, OP654796–OP654806, OP750178–OP750189.
