# Supplementary material for: Unraveling the Genetic Diversity and Phylogeography of the “King of Vitamin C” Fruit (Rosa roxburghii Trattinnick) in Chinese Southwest
Source: Ecol Evol. 2025 May 7;15(5):e71369. doi: 10.1002/ece3.71369 (PMC12058451; doi:10.1002/ece3.71369)
Supplement: Supplementary file 1 — Appendix S1. [file ECE3-15-e71369-s001.docx]

Supplementary Appendix 1. The ISSR primer information and amplification results of *Rosa roxburghii*, along with the primer information for cpDNA and SCNG sequences

|  | Primer | motif and sequence information | Annealing temperature(Tm/℃) | No. of amplified loci | No. of polymorp-hic bands | Percent of  polymorphic  bands (%) |
| --- | --- | --- | --- | --- | --- | --- |
| ISSR | 810 | (GA)8T | 49 | 11 | 11 | 100 |
|  | 827 | (AC)8G | 50 | 10 | 10 | 100 |
|  | 834 | (AG)8YT | 52 | 13 | 13 | 100 |
|  | 835 | (AG)8YC | 48 | 13 | 13 | 100 |
|  | 836 | (AG)8YA | 52 | 11 | 11 | 100 |
|  | 851 | (GT)8YG | 53 | 10 | 10 | 100 |
|  | 873 | (GACA)4 | 52 | 11 | 11 | 100 |
| CpDNA | *psb*A-*trn*H | F: GTTATGCATGAACGTAATGCTC | 52 |  | | |
|  |  | R: CGCGCATGGTGGATTCACAAATC |  |  |  |  |
|  | *atp*F-*trn*H | F: ACTCGCACACACACTCCCTTTCC | 55 |  | | |
|  |  | R: CGCGCATGGTGGATTCACAAATC |  |  |  |  |
|  | *trn*L-*trn*F | F: TTATTCGATCCAATCGTACCAC | 54 |  | | |
|  |  | R: AGAAGCCATTGCAATTGCCGGAAA |  |  |  |  |
|  | *acc*D-*psa*I | F: GGTTCAAGTCCCTCTATCCCR | 55 |  | | |
|  |  | R: ATTTGAACTGGTGACACGAG |  |  |  |  |
|  | *trn*G-*trn*S | F: GAATCGAACCCGCATCGTTAG | 54 |  | | |
|  |  | R: AACTCGTACAACGGATTAGCAATC |  |  |  |  |
| SCNG | ncpGS | F: GATGCTCACTACAAGGCTTG | 51 |  | | |
|  |  | R: AATGTGCTCTTTGTGGCGAAG |  |  |  |  |
|  | GAPDH | F: GATAGATTTGGAATTGTTGAGG | 57 |  | | |
|  |  | R: GACATTGAATGAGATAAACC |  |  |  |  |

Supplementary Appendix 2. Genetic distance (above diagonal) and genetic consistency (below diagonal) of *R. roxburghii*

| Pop. | QXS | JSX | DFX | NYX | SCX | PX | XYS | ALX | ZFX | ZYX | PDX | ZJX | PBX | GYS | LLX | TRS | XSX | BJS | ZYS | | MTX | SMX | CQS | QJS | DLS | LSX | YCS | HPX | MNX |
| --- | --- | --- | --- | --- | --- | --- | --- | --- | --- | --- | --- | --- | --- | --- | --- | --- | --- | --- | --- | --- | --- | --- | --- | --- | --- | --- | --- | --- | --- |
| QXS | - | 0.0932 | 0.1155 | 0.1818 | 0.1049 | 0.1908 | 0.1229 | 0.1467 | 0.1754 | 0.1744 | 0.2109 | 0.2083 | 0.1760 | 0.2170 | 0.1968 | 0.2232 | 0.2810 | 0.2330 | | 0.1640 | 0.2788 | 0.1667 | 0.2187 | 0.1527 | 0.1806 | 0.1955 | 0.1908 | 0.2080 | 0.3279 |
| JSX | 0.9110 | - | 0.0899 | 0.2075 | 0.1052 | 0.1533 | 0.0942 | 0.1334 | 0.1700 | 0.1894 | 0.2415 | 0.1841 | 0.1608 | 0.1846 | 0.2058 | 0.2389 | 0.2517 | 0.2451 | | 0.1899 | 0.2724 | 0.1558 | 0.2122 | 0.1088 | 0.2204 | 0.1945 | 0.2074 | 0.2262 | 0.3182 |
| DFX | 0.8909 | 0.9140 | - | 0.1543 | 0.0724 | 0.1396 | 0.0791 | 0.0988 | 0.1123 | 0.1194 | 0.2408 | 0.2252 | 0.2053 | 0.1953 | 0.2088 | 0.3246 | 0.2240 | 0.2561 | | 0.1978 | 0.3186 | 0.1779 | 0.2215 | 0.1177 | 0.2044 | 0.1411 | 0.1441 | 0.2500 | 0.2986 |
| NYX | 0.8338 | 0.8126 | 0.8570 | - | 0.1968 | 0.2246 | 0.1556 | 0.1988 | 0.1882 | 0.1948 | 0.2615 | 0.2890 | 0.2343 | 0.2608 | 0.1863 | 0.2878 | 0.2865 | 0.2361 | | 0.1817 | 0.3020 | 0.1801 | 0.1958 | 0.1992 | 0.2461 | 0.1582 | 0.1812 | 0.3031 | 0.3202 |
| SCX | 0.9004 | 0.9002 | 0.9301 | 0.8214 | - | 0.1272 | 0.1018 | 0.1180 | 0.1452 | 0.1077 | 0.2696 | 0.2399 | 0.2075 | 0.2260 | 0.1794 | 0.3184 | 0.2537 | 0.2809 | | 0.1734 | 0.3600 | 0.1687 | 0.1872 | 0.1276 | 0.1988 | 0.1579 | 0.1332 | 0.2330 | 0.2943 |
| PX | 0.8263 | 0.8579 | 0.8697 | 0.7988 | 0.8806 | - | 0.1414 | 0.1344 | 0.1089 | 0.1709 | 0.3129 | 0.2822 | 0.2321 | 0.2391 | 0.2176 | 0.2966 | 0.2505 | 0.3156 | | 0.2085 | 0.2953 | 0.1892 | 0.2258 | 0.1295 | 0.2084 | 0.1642 | 0.1379 | 0.2479 | 0.3635 |
| XYS | 0.8844 | 0.9101 | 0.9239 | 0.8559 | 0.9032 | 0.8682 | - | **0.0653** | 0.1062 | 0.1310 | 0.2541 | 0.2513 | 0.2151 | 0.1950 | 0.2080 | 0.2775 | 0.2757 | 0.2767 | | 0.1726 | 0.2724 | 0.1644 | 0.2309 | 0.1099 | 0.2372 | 0.1463 | 0.1448 | 0.2172 | 0.3411 |
| ALX | 0.8636 | 0.8751 | 0.9059 | 0.8197 | 0.8887 | 0.8743 | **0.9367** | - | 0.1034 | 0.1459 | 0.2818 | 0.2544 | 0.2545 | 0.2062 | 0.2433 | 0.3234 | 0.3065 | 0.3169 | | 0.2168 | 0.2780 | 0.2103 | 0.2433 | 0.1215 | 0.2349 | 0.1453 | 0.1204 | 0.2314 | 0.3130 |
| ZFX | 0.8392 | 0.8436 | 0.8938 | 0.8284 | 0.8648 | 0.8968 | 0.8993 | 0.9018 | - | 0.1462 | 0.2934 | 0.2853 | 0.2762 | 0.2861 | 0.2351 | 0.4001 | 0.2772 | 0.3074 | | 0.1839 | 0.3294 | 0.2208 | 0.2372 | 0.1618 | 0.2404 | 0.1213 | 0.1068 | 0.2589 | 0.3196 |
| ZYX | 0.8400 | 0.8274 | 0.8875 | 0.8230 | 0.8979 | 0.8429 | 0.8772 | 0.8643 | 0.8640 | - | 0.2640 | 0.2484 | 0.1940 | 0.1675 | 0.1509 | 0.2923 | 0.2062 | 0.2968 | | 0.2225 | 0.3218 | 0.2449 | 0.1971 | 0.1853 | 0.3102 | 0.1713 | 0.1453 | 0.2359 | 0.3221 |
| PDX | 0.8098 | 0.7855 | 0.7860 | 0.7699 | 0.7637 | 0.7314 | 0.7756 | 0.7544 | 0.7457 | 0.7680 | - | 0.1435 | 0.1783 | 0.1925 | 0.2559 | 0.1917 | 0.2446 | 0.2025 | | 0.2249 | 0.2316 | 0.1770 | 0.1756 | 0.2439 | 0.2727 | 0.3041 | 0.2645 | 0.2483 | 0.3354 |
| ZJX | 0.8120 | 0.8319 | 0.7983 | 0.7490 | 0.7867 | 0.7542 | 0.7778 | 0.7754 | 0.7518 | 0.7801 | 0.8663 | - | 0.1252 | 0.2346 | 0.1839 | 0.2335 | 0.2122 | 0.2835 | | 0.2698 | 0.2683 | 0.1730 | 0.2626 | 0.2487 | 0.3516 | 0.3176 | 0.3029 | 0.3255 | 0.3860 |
| PBX | 0.8386 | 0.8515 | 0.8144 | 0.7911 | 0.8126 | 0.7929 | 0.8064 | 0.7753 | 0.7587 | 0.8236 | 0.8367 | 0.8823 | - | 0.1841 | 0.1215 | 0.1943 | 0.1533 | 0.2663 | | 0.2318 | 0.2543 | 0.1874 | 0.2625 | 0.2475 | 0.2921 | 0.2758 | 0.2975 | 0.2643 | 0.4513 |
| GYS | 0.8049 | 0.8315 | 0.8226 | 0.7704 | 0.7977 | 0.7873 | 0.8228 | 0.8137 | 0.7512 | 0.8458 | 0.8249 | 0.7909 | 0.8319 | - | 0.2041 | 0.1578 | 0.2377 | 0.3120 | | 0.3521 | 0.2372 | 0.2402 | 0.2006 | 0.1752 | 0.2828 | 0.2828 | 0.2680 | 0.2644 | 0.3610 |
| LLX | 0.8213 | 0.8140 | 0.8115 | 0.8300 | 0.8357 | 0.8045 | 0.8122 | 0.7840 | 0.7905 | 0.8599 | 0.7742 | 0.8321 | 0.8856 | 0.8154 | - | 0.1464 | 0.1456 | 0.2643 | | 0.2416 | 0.2855 | 0.2084 | 0.2623 | 0.2337 | 0.3045 | 0.2325 | 0.2360 | 0.3008 | 0.4242 |
| TRS | 0.7999 | 0.7875 | 0.7228 | 0.7499 | 0.7273 | 0.7434 | 0.7576 | 0.7237 | 0.6703 | 0.7466 | 0.8256 | 0.7918 | 0.8234 | 0.8540 | 0.8638 | - | 0.2380 | 0.2512 | | 0.2959 | 0.1628 | 0.2069 | 0.2496 | 0.2511 | 0.2704 | 0.3487 | 0.3029 | 0.2529 | 0.4554 |
| XSX | 0.7550 | 0.7775 | 0.7993 | 0.7509 | 0.7759 | 0.7784 | 0.7590 | 0.7360 | 0.7579 | 0.8137 | 0.7830 | 0.8088 | 0.8578 | 0.7884 | 0.8645 | 0.7882 | - | 0.2125 | | 0.2955 | 0.2442 | 0.2612 | 0.3308 | 0.2860 | 0.3305 | 0.2891 | 0.2431 | 0.3217 | 0.4688 |
| BJS | 0.7922 | 0.7826 | 0.7740 | 0.7897 | 0.7551 | 0.7294 | 0.7583 | 0.7284 | 0.7353 | 0.7432 | 0.8167 | 0.7531 | 0.7662 | 0.7320 | 0.7678 | 0.7779 | 0.8086 | - | | 0.2937 | 0.3043 | 0.2838 | 0.2881 | 0.2499 | 0.3062 | 0.3097 | 0.2339 | 0.2772 | 0.3429 |
| ZYS | 0.8487 | 0.8271 | 0.8205 | 0.8339 | 0.8408 | 0.8118 | 0.8414 | 0.8051 | 0.8320 | 0.8005 | 0.7986 | 0.7635 | 0.7931 | 0.7032 | 0.7854 | 0.7439 | 0.7442 | 0.7455 | | - | 0.3156 | 0.2173 | 0.1991 | 0.2277 | 0.2127 | 0.1940 | 0.1479 | 0.2393 | 0.3717 |
| MTX | 0.7567 | 0.7616 | 0.7272 | 0.7393 | 0.6976 | 0.7443 | 0.7615 | 0.7573 | 0.7194 | 0.7248 | 0.7932 | 0.7647 | 0.7755 | 0.7888 | 0.7516 | 0.8498 | 0.7833 | 0.7376 | | 0.7294 | - | 0.1955 | 0.3280 | 0.2725 | 0.2928 | 0.2801 | 0.2620 | 0.2932 | **0.4762** |
| SMX | 0.8464 | 0.8557 | 0.8370 | 0.8352 | 0.8448 | 0.8276 | 0.8484 | 0.8104 | 0.8019 | 0.7828 | 0.8378 | 0.8411 | 0.8291 | 0.7864 | 0.8119 | 0.8131 | 0.7701 | 0.7529 | | 0.8047 | 0.8224 | - | 0.1648 | 0.1726 | 0.2453 | 0.2250 | 0.2084 | 0.2612 | 0.4696 |
| CQS | 0.8036 | 0.8088 | 0.8013 | 0.8221 | 0.8293 | 0.7979 | 0.7938 | 0.7840 | 0.7888 | 0.8211 | 0.8389 | 0.7690 | 0.7691 | 0.8183 | 0.7693 | 0.7791 | 0.7183 | 0.7497 | | 0.8195 | 0.7203 | 0.8481 | - | 0.1675 | 0.2631 | 0.2178 | 0.1707 | 0.2353 | 0.2643 |
| QJS | 0.8584 | 0.8969 | 0.8890 | 0.8194 | 0.8802 | 0.8785 | 0.8960 | 0.8855 | 0.8506 | 0.8309 | 0.7836 | 0.7798 | 0.7807 | 0.8393 | 0.7916 | 0.7779 | 0.7513 | 0.7789 | | 0.7963 | 0.7615 | 0.8415 | 0.8458 | - | 0.1788 | 0.1723 | 0.1067 | 0.2226 | 0.2624 |
| DLS | 0.8348 | 0.8022 | 0.8151 | 0.7818 | 0.8197 | 0.8119 | 0.7888 | 0.7907 | 0.7863 | 0.7333 | 0.7614 | 0.7036 | 0.7467 | 0.7537 | 0.7375 | 0.7630 | 0.7186 | 0.7362 | | 0.8084 | 0.7462 | 0.7824 | 0.7686 | 0.8363 | - | 0.1863 | 0.1586 | 0.2183 | 0.3267 |
| LSX | 0.8224 | 0.8233 | 0.8684 | 0.8537 | 0.8539 | 0.8486 | 0.8639 | 0.8647 | 0.8857 | 0.8425 | 0.7378 | 0.7279 | 0.7590 | 0.7537 | 0.7926 | 0.7056 | 0.7489 | 0.7337 | | 0.8237 | 0.7557 | 0.7985 | 0.8043 | 0.8417 | 0.8300 | - | 0.0918 | 0.2037 | 0.2688 |
| YCS | 0.8263 | 0.8127 | 0.8658 | 0.8342 | 0.8753 | 0.8712 | 0.8652 | 0.8865 | 0.8987 | 0.8648 | 0.7676 | 0.7386 | 0.7427 | 0.7649 | 0.7898 | 0.7387 | 0.7842 | 0.7914 | | 0.8625 | 0.7695 | 0.8119 | 0.8431 | 0.8988 | 0.8533 | 0.9123 | - | 0.1836 | 0.2695 |
| HPX | 0.8122 | 0.7976 | 0.7788 | 0.7385 | 0.7922 | 0.7804 | 0.8047 | 0.7935 | 0.7719 | 0.7898 | 0.7801 | 0.7221 | 0.7678 | 0.7677 | 0.7403 | 0.7765 | 0.7249 | 0.7579 | | 0.7872 | 0.7459 | 0.7702 | 0.7903 | 0.8004 | 0.8039 | 0.8157 | 0.8323 | - | 0.4030 |
| MNX | 0.7204 | 0.7274 | 0.7419 | 0.7260 | 0.7451 | 0.6952 | 0.7110 | 0.7312 | 0.7264 | 0.7246 | 0.7151 | 0.6798 | 0.6368 | 0.6970 | 0.6543 | 0.6342 | 0.6257 | 0.7097 | | 0.6896 | 0.6211 | 0.6252 | 0.7678 | 0.7692 | 0.7213 | 0.7643 | 0.7638 | 0.6683 | - |

**
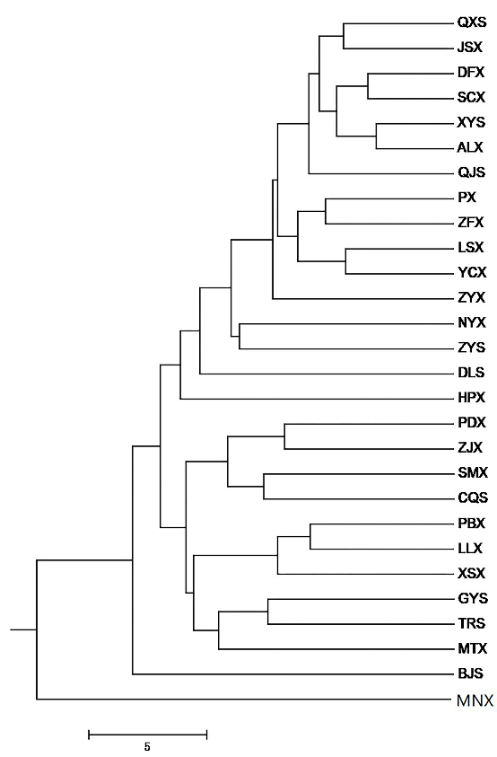
**

Supplementary Appendix 3. Phylogenetic tree of NJ constructed

based on ISSR of *R. roxburghii* populations


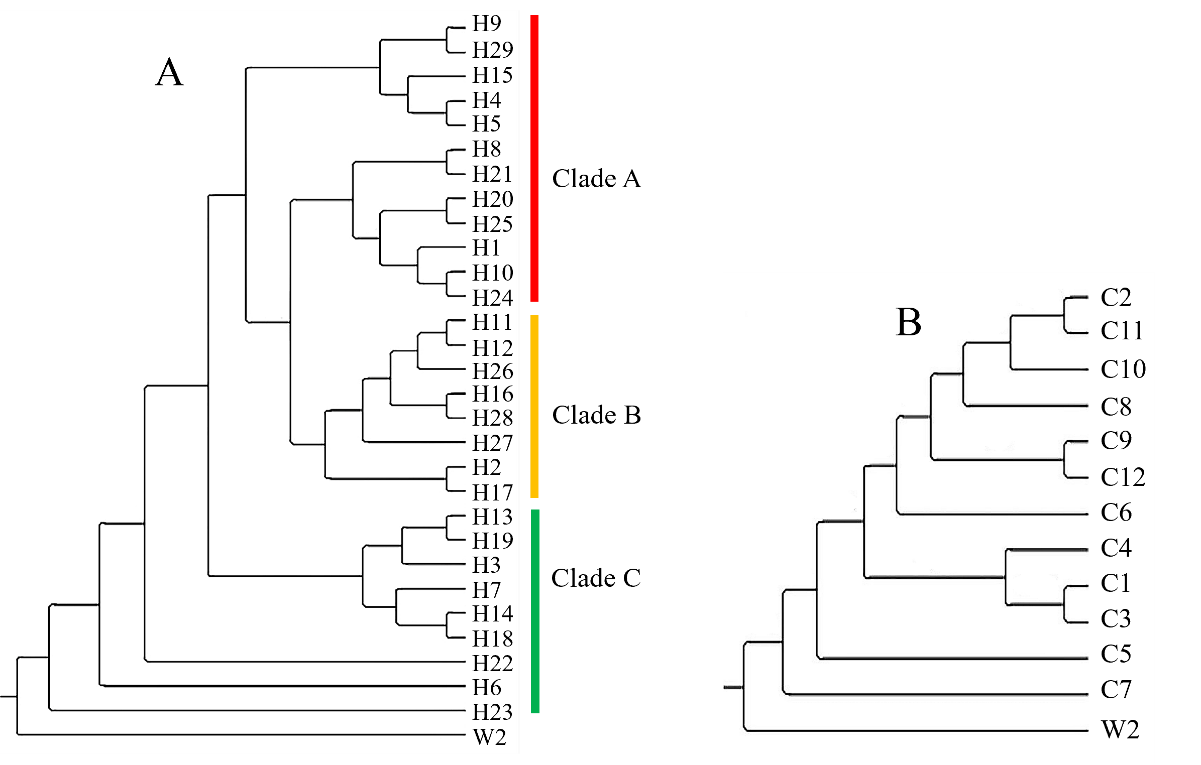


Supplementary Appendix 4. Phylogenetic trees of chloroplast gene haplotypes (A) and single-copy nuclear gene haplotypes (B) constructed using the maximum likelihood method (ML)

**
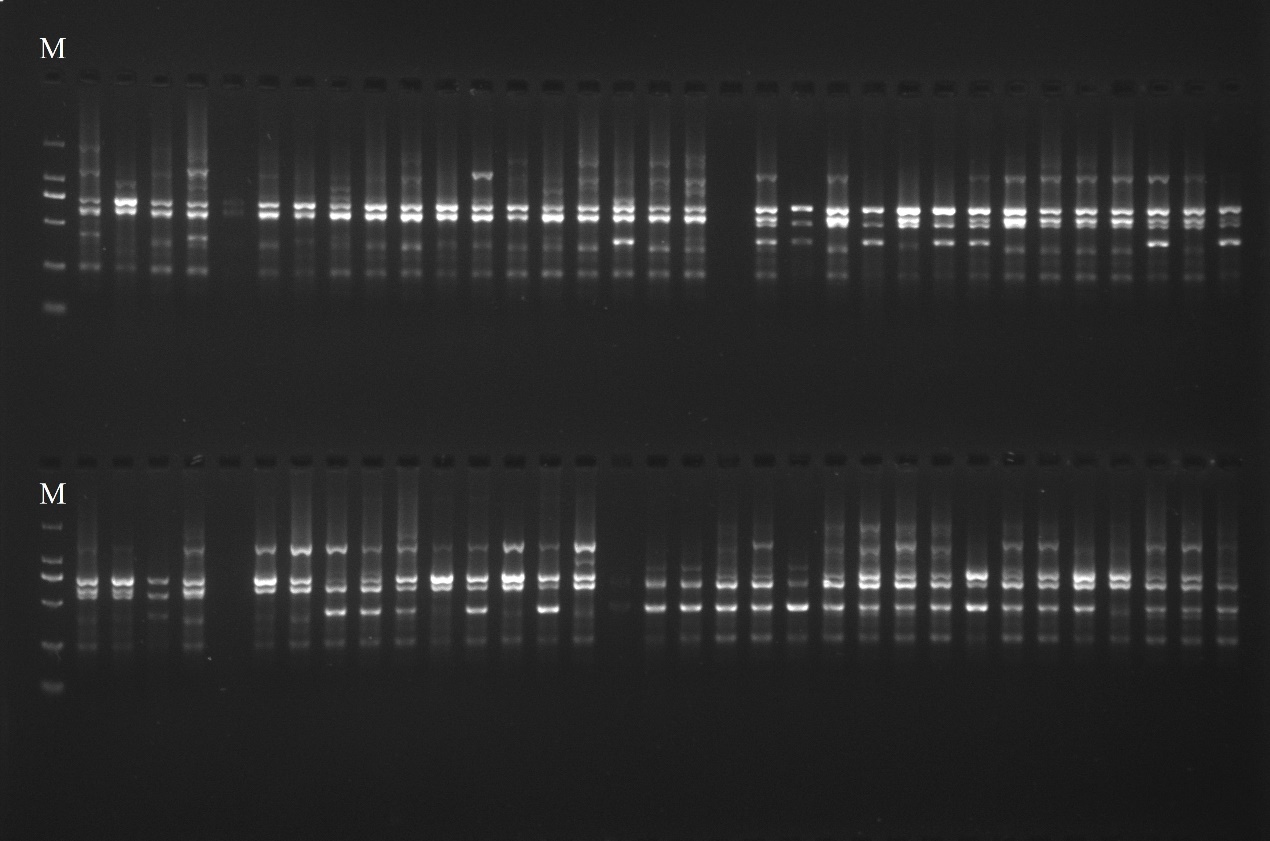
**

1. Primer 810 amplification results. M: Marker (DL 2000)

**
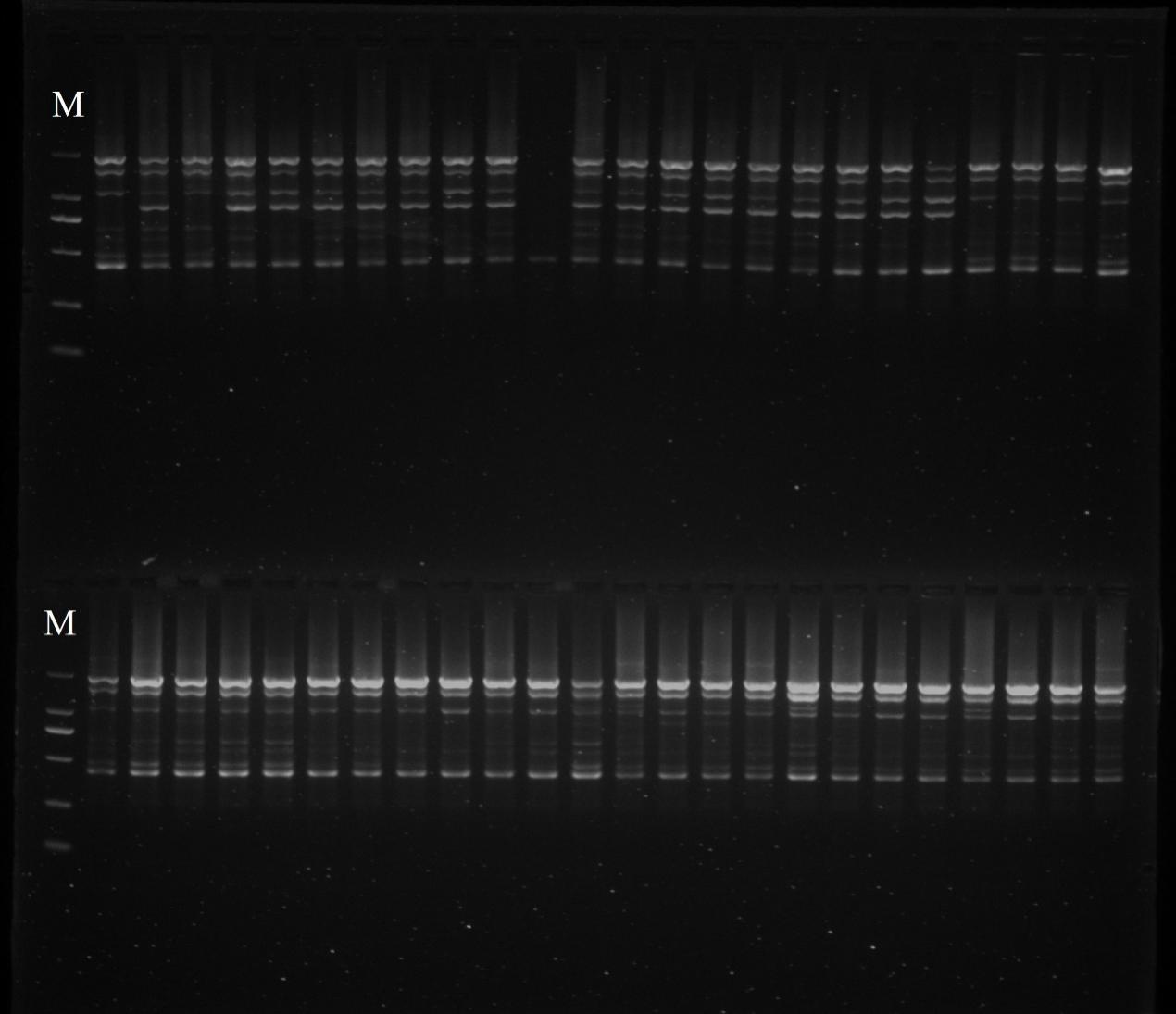
**

1. Primer 827 amplification results. M: Marker (DL 2000)

**
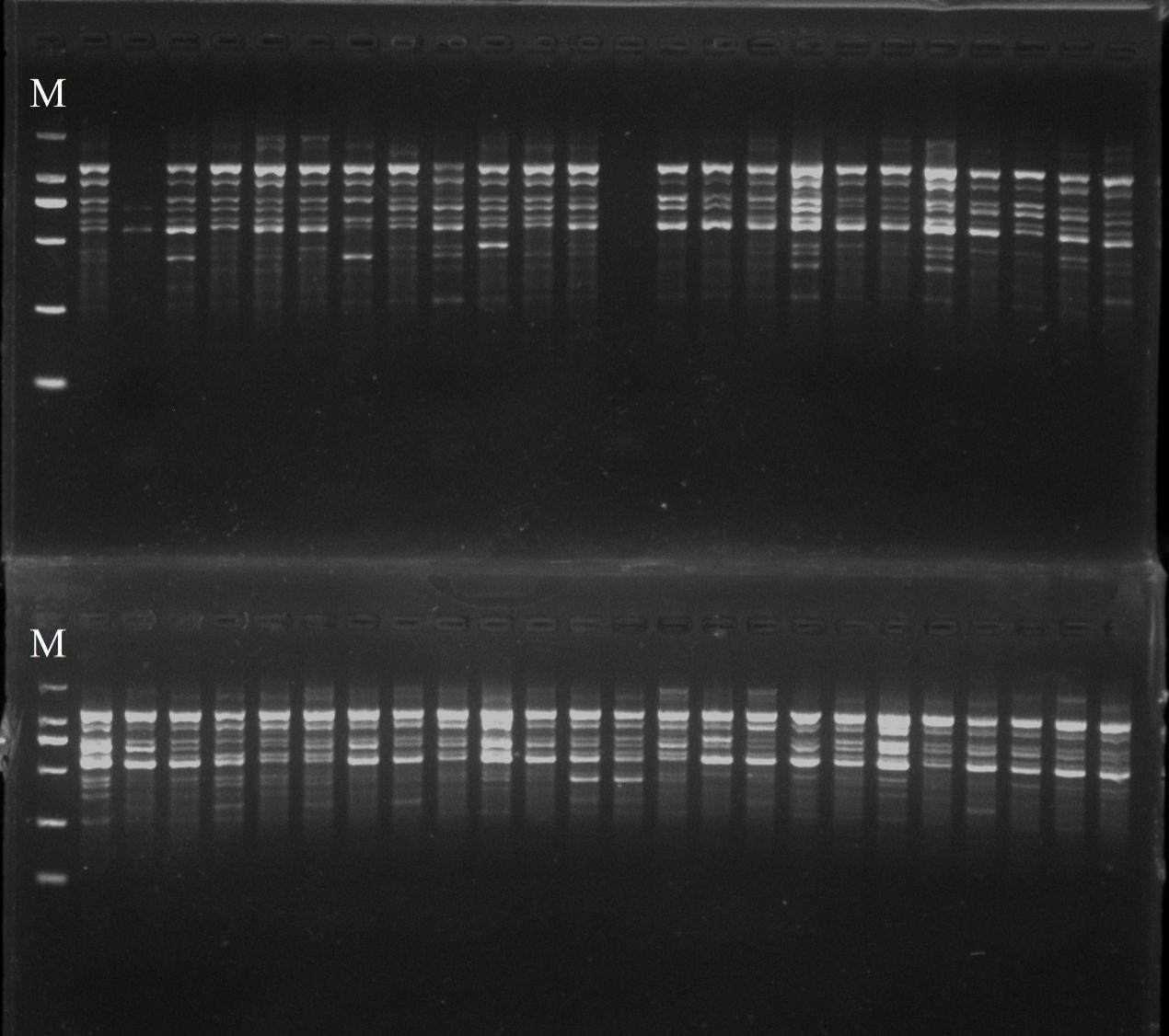
**

1. Primer 834 amplification results. M: Marker (DL 2000)

**
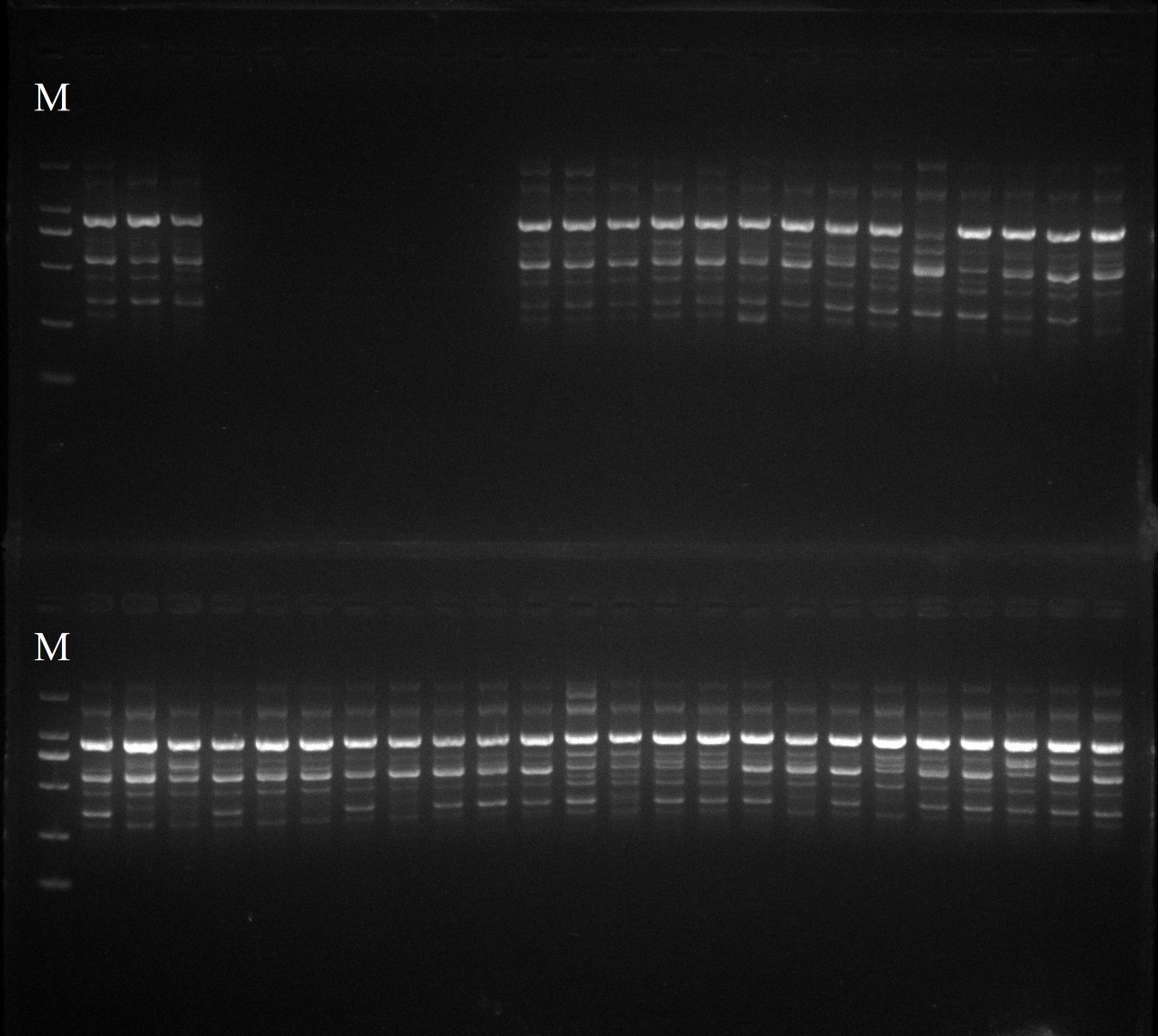
**

1. Primer 835 amplification results. M: Marker (DL 2000)

**
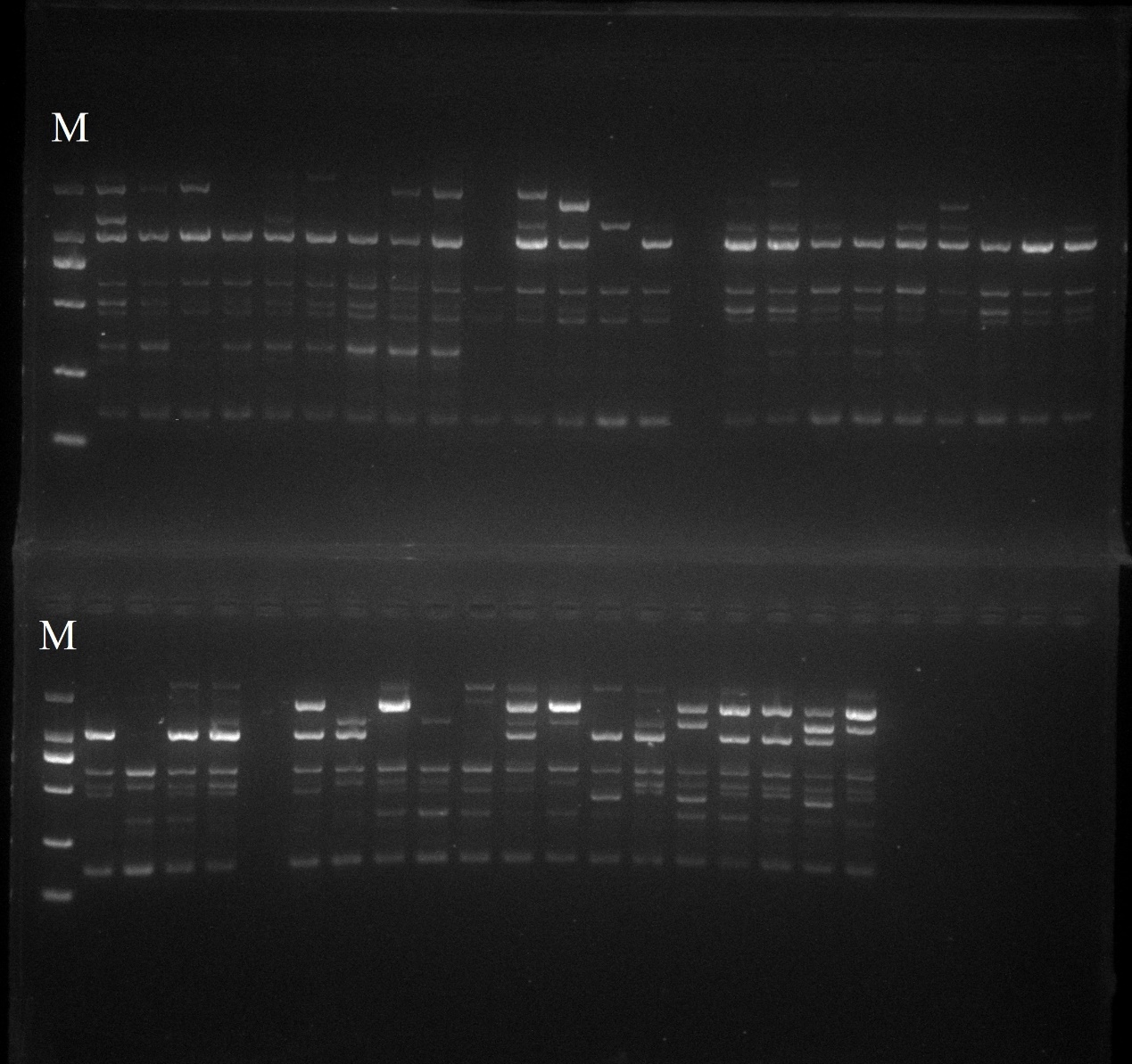
**

1. Primer 836 amplification results. M: Marker (DL 2000)

**
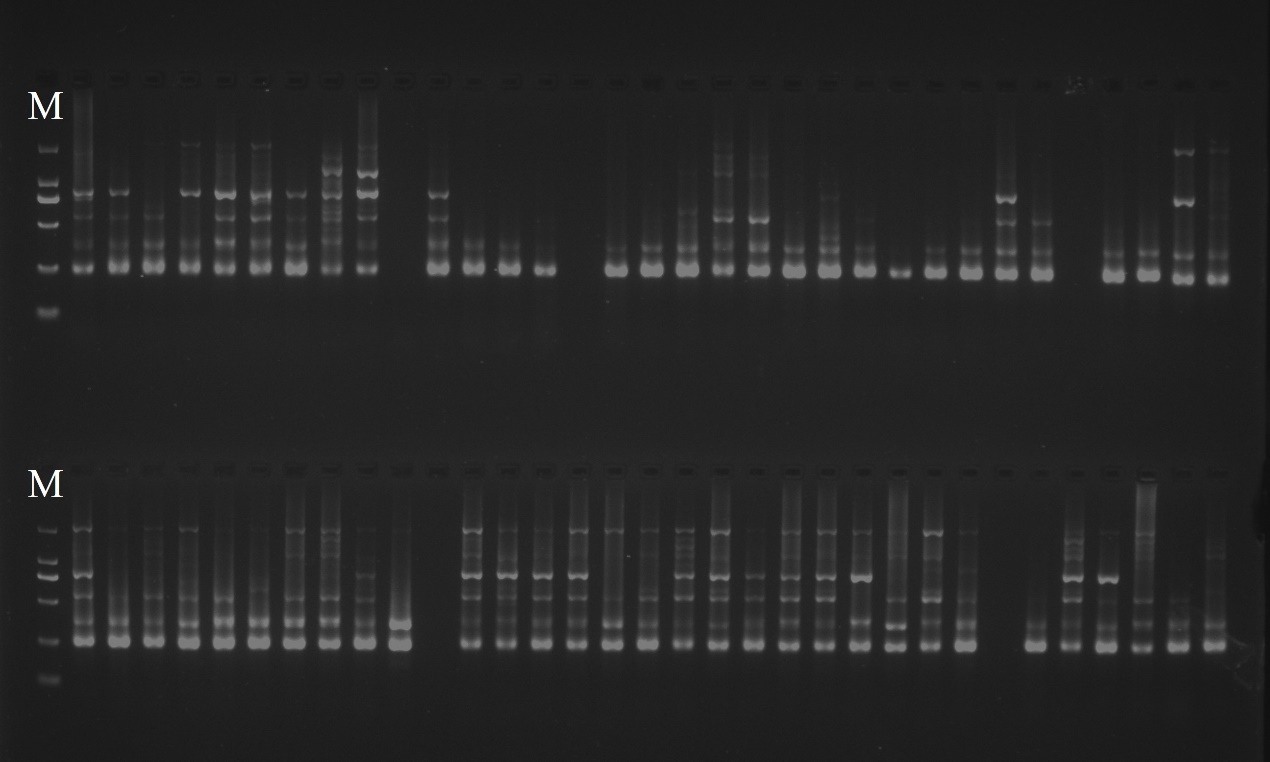
**

1. Primer 851 amplification results. M: Marker (DL 2000)

**
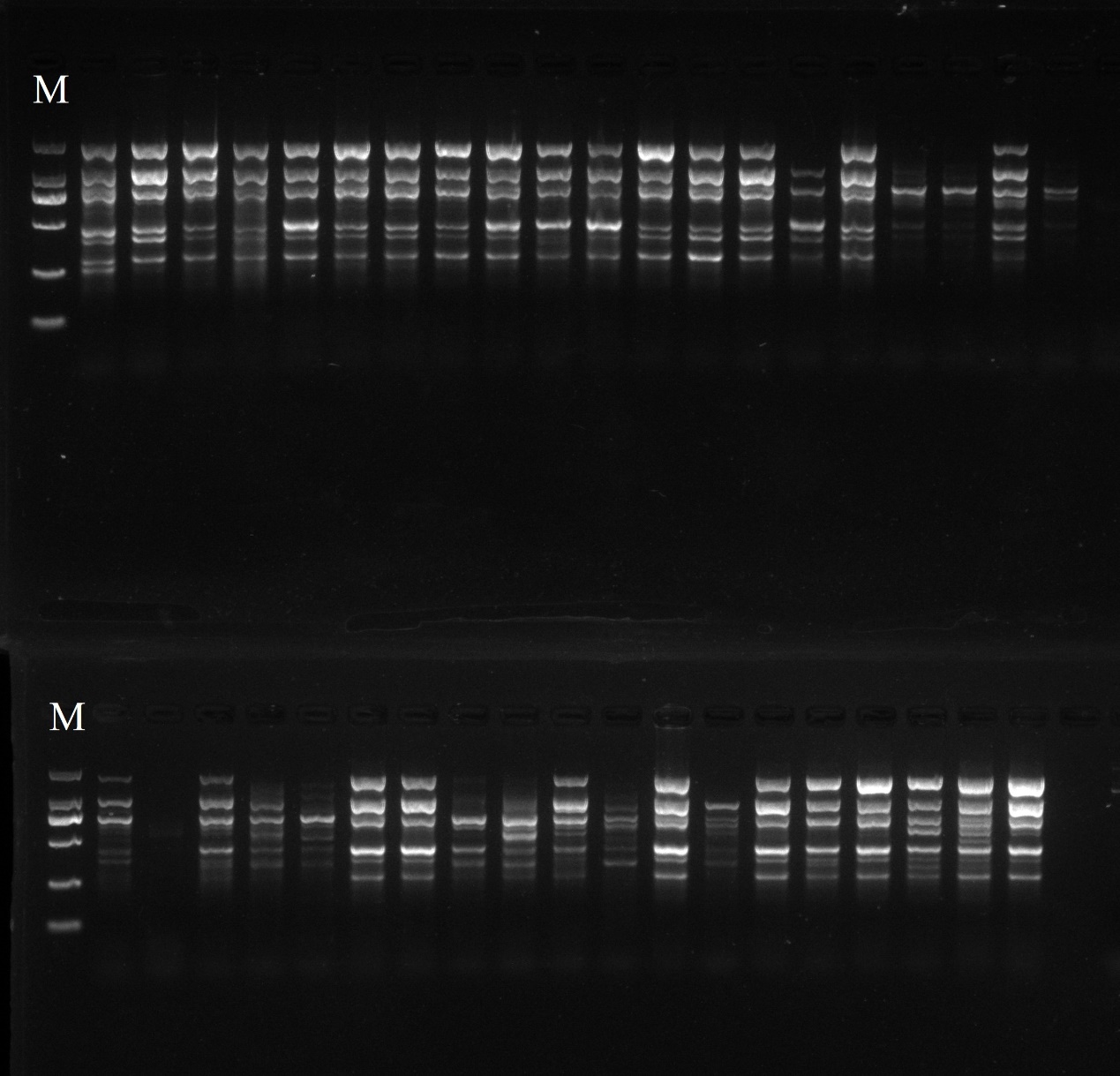
**

1. Primer 873 amplification results. M: Marker (DL 2000)

Supplementary Appendix 5. The gel electrophoresis map of 7 ISSR markers
